# Toward a gold standard for benchmarking gene set enrichment analysis

**DOI:** 10.1093/bib/bbz158

**Published:** 2020-03-09

**Authors:** Ludwig Geistlinger, Gergely Csaba, Mara Santarelli, Marcel Ramos, Lucas Schiffer, Nitesh Turaga, Charity Law, Sean Davis, Vincent Carey, Martin Morgan, Ralf Zimmer, Levi Waldron

**Affiliations:** 1 Graduate School of Public Health and Health Policy, City University of New York, New York, NY 10027, USA; 2 Institute for Implementation Science and Population Health, City University of New York, New York, NY 10027, USA; 3 Institute for Bioinformatics, Ludwig-Maximilians-Universität München, 80333 Munich, Germany; 4 Roswell Park Cancer Institute, Buffalo, NY 14203, USA; 5 Graduate School of Arts and Sciences, Boston University, Boston, MA 02215, USA; 6 Epigenetics and Development Division, The Walter and Eliza Hall Institute of Medical Research, Parkville, Victoria 3052, Australia; 7 Department of Medical Biology, The University of Melbourne, Parkville, Victoria 3010, Australia; 8 Center for Cancer Research, National Cancer Institute, Bethesda, MD 20892, USA; 9 Harvard Medical School, Boston, MA 02215, USA

**Keywords:** gene set analysis, pathway analysis, gene expression data, microarray, RNA-seq

## Abstract

**Motivation:**

Although gene set enrichment analysis has become an integral part of high-throughput gene expression data analysis, the assessment of enrichment methods remains rudimentary and *ad hoc*. In the absence of suitable gold standards, evaluations are commonly restricted to selected datasets and biological reasoning on the relevance of resulting enriched gene sets.

**Results:**

We develop an extensible framework for reproducible benchmarking of enrichment methods based on defined criteria for applicability, gene set prioritization and detection of relevant processes. This framework incorporates a curated compendium of 75 expression datasets investigating 42 human diseases. The compendium features microarray and RNA-seq measurements, and each dataset is associated with a precompiled GO/KEGG relevance ranking for the corresponding disease under investigation. We perform a comprehensive assessment of 10 major enrichment methods, identifying significant differences in runtime and applicability to RNA-seq data, fraction of enriched gene sets depending on the null hypothesis tested and recovery of the predefined relevance rankings. We make practical recommendations on how methods originally developed for microarray data can efficiently be applied to RNA-seq data, how to interpret results depending on the type of gene set test conducted and which methods are best suited to effectively prioritize gene sets with high phenotype relevance.

**Availability:**

http://bioconductor.org/packages/GSEABenchmarkeR

**Contact:**

ludwig.geistlinger@sph.cuny.edu

## 1 Introduction

The goal of genome-wide gene expression studies is to discover the molecular mechanisms that underlie certain phenotypes such as human diseases [1]. For this purpose, expression changes of individual genes are typically analyzed for enrichment in functional gene sets. These sets may represent molecular functions and biological processes as defined by the Gene Ontology (GO) [2], pathway databases such as KEGG [3] and Reactome [4] or experimentally derived gene sets such as available in the MSigDB [5]. The two predominantly used enrichment methods are (i) overrepresentation analysis (ORA), testing whether a gene set contains disproportionately many genes of significant expression change, and (ii) gene set enrichment analysis [7, GSEA], rather testing whether genes of a gene set accumulate at the top or bottom of the full gene vector ordered by direction and magnitude of expression change. Both methods are the foundation of many popular enrichment tools including DAVID [8], Enrichr [9] and clusterProfiler [10]. However, the term GSEA now encompasses a general strategy implemented by a wide range of methods [11]. Those methods share a common goal, although approaches and statistical models vary substantially. There are various ways by which the existing methods can be categorized. In their seminal paper, Goeman and Bühlmann [6] categorize enrichment methods based on the underlying null hypothesis as ‘competitive’ or ‘self-contained’. A ‘competitive’ method compares a gene set against the background of all genes not in the set, assessing whether the level of differential expression (DE) in the gene set exceeds the background level. A ‘self-contained’ method analyzes each gene set in isolation, assessing DE of the gene set without comparing to a background [12, for a review]. Khatri *et al*. [13] took a different approach by dividing methods along the timeline of development into three generations: (i) ‘overrepresentation’ methods such as ORA, which first reduce the full expression matrix to genes passing a threshold for DE, and subsequently concentrate analysis on the list of differentially expressed genes, (ii) ‘gene set scoring’ methods such as GSEA, which first compute DE scores for all genes measured, and subsequently compute gene set scores by aggregating the scores of contained genes and (iii) ‘network-based’ methods, which evaluate measures of DE in the context of known interactions between genes as defined in signaling pathways and gene regulatory networks [14]. Methods can be further categorized based on whether they test a ‘directional’ hypothesis (genes in the set tend to be either predominantly up- or down-regulated) or a ‘mixed’ hypothesis (genes in the set tend to be differentially expressed, regardless of the direction); whether they focus on binary case-control comparisons or also support more complex experimental designs; and, relatedly, whether they analyze expression differences of gene sets between sample groups or whether they score gene set activity levels for single samples [15, 16]. Given the variety of existing methods with individual benefits and limitations, a major question is thus which method is best suited for the enrichment analysis (EA). As a consequence, many methods have been published claiming improvement, especially with respect to ORA and the original GSEA method. This claim is typically made based on (i) simulated data, specifically designed to demonstrate beneficial aspects of a new method, and (ii) experimental datasets, for which however the truly enriched gene sets are not known. As the evaluation is thus typically based on self-defined standards including only a few methods, Mitrea *et al*. [17] identified the lack of gold standards for consistent assessment and comparison of enrichment methods as a major bottleneck. Steps toward an objective assessment are recent independent studies [18–22], which evaluated a partly overlapping selection of enrichment methods on (i) simulated data, modeling certain aspects of experimental data [18]; (ii) experimental data, arguing on the biological relevance of the enriched gene sets [19]; or (iii) a combination of both data types [20–23]. As the standard of truth is hard to establish for experimental data, several approaches have been suggested to a priori define target gene sets for specific datasets. For example, Naeem *et al*. [24] suggested an assessment based on known target gene sets of transcription factors for expression datasets where those transcription factors are overexpressed or knocked out as available for *Escherichia coli* and *Saccharomyces cerevisiae*. On the other hand, Tarca *et al*. [25, 26] collected 42 microarray datasets investigating human diseases for which a specific KEGG target pathway exists. This strategy has been adapted by several recent enrichment evaluation studies [27–31]. However, there is little agreement among studies on which methods to prefer, with most studies concluding with a recommendation for a consensus/combination of methods [21, 23, 24, 29]. Although this is valuable in practice, existing assessments (i) were mostly based on microarray data, and it is not clear whether results hold equally for RNA-seq data, (ii) do not represent the wide range of existing methods and (iii) are often cumbersome to reproduce for additional methods, as this involves considerable effort of data processing and method collection.

## 2 Methods

### 2.1 Construction of the benchmark compendia

**Figure 1:**
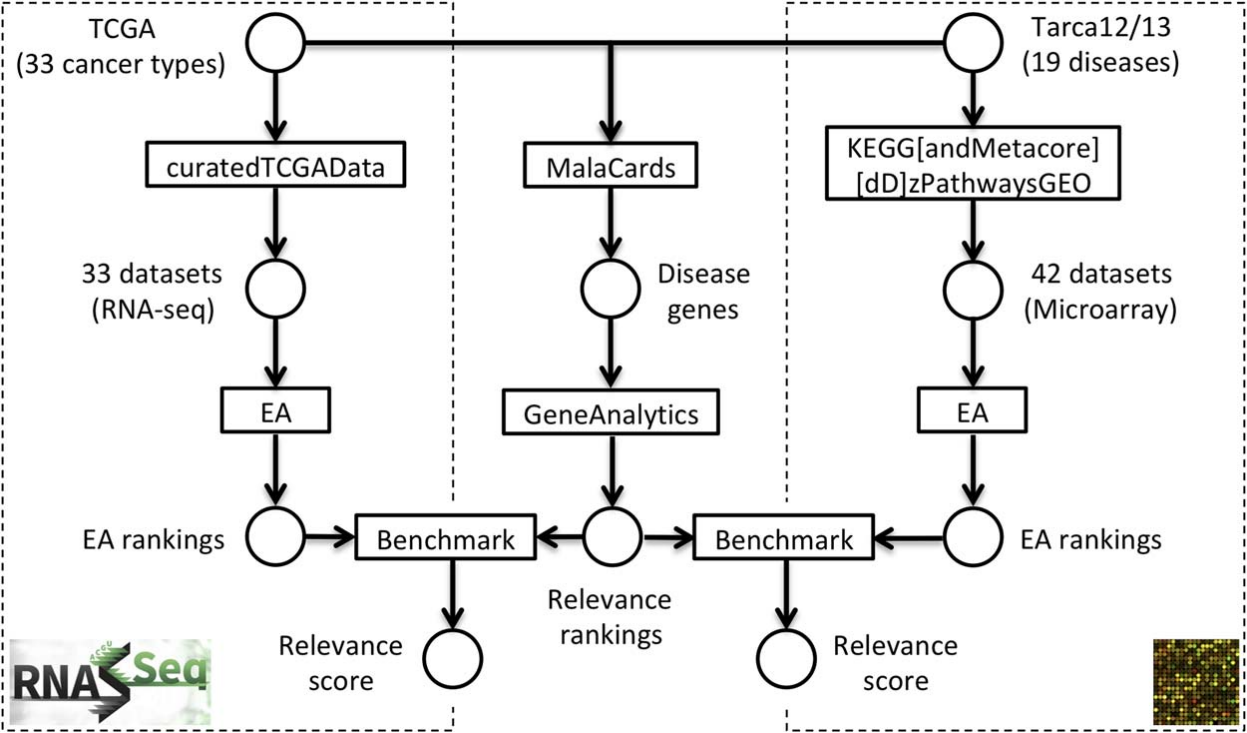
Benchmark setup. The benchmark framework incorporates a pre-defined RNA-seq panel (left), gene set relevance rankings (center) and a microarray panel (right). The RNA-seq panel investigates 33 cancer types across 33 datasets from TCGA [32], which are accessed through the curatedTCGAData package. The microarray panel investigates 19 human diseases across 42 datasets collected by Tarca et al. [25, 26], which are available in the KEGGdzPathwaysGEO and KEGGandMetacoreDzPathwaysGEO packages. Gene set relevance rankings for both data panels are constructed by (i) querying the MalaCards database [33] for each disease investigated and (ii) subjecting resulting disease genes to GeneAnalytics [34], which yields relevance rankings for GO-BP terms and KEGG pathways. EA methods selected for benchmarking are carried out across datasets of the data panels, yielding a gene set ranking (EA ranking) for each method on each dataset. The resulting EA rankings for each dataset are then benchmarked against the precompiled relevance rankings for the corresponding disease investigated.

As illustrated in Figure 1, the two pre-defined benchmark compendia consist of 42 microarray datasets collected by Tarca *et al*. [25, 26, GEO2KEGG] and 33 RNA-seq datasets from The Cancer Genome Atlas [32, TCGA]. These datasets investigate 42 human diseases, including 35 cancer types (Supplementary Tables S1 and S2). Gene set relevance rankings for each disease were constructed by querying the MalaCards database [33]. MalaCards scores genes for disease relevance based on experimental evidence and co-citation in the literature. Per-gene relevance was summarized across GO and KEGG gene sets by subjecting disease-relevant genes for each disease to the GeneAnalytics [34] web tool. GeneAnalytics computes composite relevance scores for each gene set based on the relevance scores of the contained genes, weighted by the proportion of relevant genes and the number of data sources supporting the relevance of genes in the gene set (Supplementary Methods S1.3).

**Table 1:** Gene set analysis methods under benchmark

**Method**	**Author**	**Year**	**Citations** $^1$	**RNA-seq**	**Gene statistic** $^2$	**Set statistic**	**Significance estimation**
ORA	–$^3$	–$^3$	–$^3$	✓	user-defined	DE / GS overlap	Fisher’s exact test
GLOBALTEST	Goeman *et al*. [68]	2004	983	–	–	$Q$ statistic	Empirical Bayes GLM
GSEA	Subramanian *et al*. [7]	2005	16 730	–	$t_{\text{S2N}}$	KS statistic	Sample permutation
SAFE	Barry *et al*. [54]	2005	350	–	Student’s $t$	Wilcoxon rank sum	Sample permutation
GSA	Efron and Tibshirani [62]	2007	798	–	$t_{\textrm{SAM}}$	Maxmean	Sample permutation
SAMGS	Dinu *et al*. [69]	2007	270	–	$t_{\textrm{SAM}}$	Hotelling’s $T^2$	Sample permutation
ROAST	Wu *et al*. [70]	2010	253	✓	$t_{\textrm{MOD}}$	Weighted mean	Rotation
CAMERA	Wu and Smyth [66]	2012	246	✓	$t_{\textrm{MOD}}$	$t_{\textrm{IGC}}$	Two-sample $t$-test
PADOG	Tarca *et al*. [25]	2012	71	–	$|t_{\textrm{MOD}}|$	Weighted mean	Sample permutation
GSVA	Hänzelmann *et al*. [71]	2013	471	✓	–	KS statistic	Empirical Bayes GLM

See Supplementary Table S3 for additional methodological differences including directionality, supported experimental designs and whether a pre-ranked execution mode is available. Abbreviations: DE, differential expression; GS, gene set; KS, Kolmogorov–Smirnov; GLM, generalized linear model.^1^Google Scholar, May 2019.^2^Notation for specific modifications of the regular $t$-statistic: $t$-like signal-to-noise ratio $t_{\text{S2N}}$ [59]; SAM’s $t_{\textrm{SAM}}$ accounting for small variability at low expression levels [72]; moderated $t$-statistic $t_{\textrm{MOD}}$ [38]; and extended $t$-statistic accounting for inter-gene correlation $t_{\textrm{IGC}}$ [66].^3^Popular implementations are available in DAVID [8] and PathwayStudio [67] among many others [11].

### 2.2 Enrichment methods

Enrichment methods selected for assessment are listed in Table 1. Methods were carried out as implemented in the EnrichmentBrowser package [29]. See Supplementary Methods S1.1 for an overview of main features and implementation details of each method. Sample permutation methods originally developed for microarray data were assessed in two different ways on RNA-seq data (see column ‘RNA-seq’ in Table 1). As these methods compute $t$-like statistics for each gene in each permutation of the sample labels, we (i) carried these methods out after applying a variance-stabilizing transformation (VST) or (ii) adapted methods to employ RNA-seq tools for computation of the per-gene DE statistic in each permutation. For the VST we used the cpm function implemented in the edgeR package [35] to compute moderated log2 read counts. Using edgeR’s estimate of the common dispersion $\phi $, the prior.count parameter of the cpm function was chosen as $0.5/\phi $ as previously suggested [36, 37]. On the other hand, methods were adapted as previously described [29] to use limma/voom [38, 39], edgeR or DESeq2 [40] for computation of the per-gene statistic in each permutation of sample labels.

### 2.3 Gene set collections

Gene set collections were defined according to human KEGG pathways and GO terms of the biological process (GO-BP) ontology using the function getGenesets from the EnrichmentBrowser package. Collections were restricted to gene sets with a minimum and maximum size of 5 and 500, respectively. This yielded 323 KEGG gene sets and 4631 GO-BP gene sets with a median gene set size of 72 and 11, respectively.

### 2.4 Runtime

Elapsed runtime was evaluated using the R function system.time on an Intel Xeon 2.7 GHz machine.

### 2.5 Statistical significance

The fraction of statistically significant gene sets returned by an EA method when applied to a specific dataset was evaluated with and without multiple testing correction. A nominal significance level of 0.05 was used when not correcting for multiple testing. Multiple testing correction was carried out using the method from Benjamini and Hochberg (BH) [41] with an FDR cutoff of 0.05. ‘Type I error rate’ was evaluated by randomization of the sample labels on the dataset from [42]. The dataset contains microarray measurements of acute myeloid leukemia (AML) and acute lymphoblastic leukemia (ALL) patients and is available from Bioconductor in the golubEsets data package [43]. Probe level measurements were normalized using the vsn2 function of the vsn package [44]. Normalized data were summarized to gene level using the probe2gene function of the EnrichmentBrowser package. The type I error rate was estimated for each enrichment method by shuffling the sample labels (ALL vs. AML) 1000 times and assessing in each permutation the fraction of gene sets with $P < 0.05$. ‘Random gene sets’ of increasing set size were analyzed to assess whether enrichment methods are affected by gene set size. We therefore sampled 100 random gene sets of defined size $s \in \{5, 10, 25, 50, 100, 250, 500\}$ and assessed the fraction of significant gene sets for each enrichment method when applied to the Golub dataset using the true sample labels.

### 2.6 Phenotype relevance

To evaluate the phenotype relevance of a gene set ranking $R_{m(d)}$ obtained from the application of an EA method $m$ to an expression dataset $d$ investigating phenotype $p$, we assess whether the ranking accumulates phenotype-relevant gene sets at the top. Therefore, we first transform the ranks from the EA to weights—where the greater the weight of a gene set, the more it is ranked toward the top of $R_{m(d)}$.

#### 2.6.1 Transformation of gene set ranks into weights

EA methods return gene sets ranked according to a ranking statistic $S$, typically the gene set $P$-value or gene set score. If the number of gene sets investigated is $N_{GS}$, then absolute ranks $r_A$ run from 1 to $N_{GS}$. Relative ranks

(1) \begin{equation*} r_R = r_A / N_{GS} \end{equation*}

can then be transformed into weights $w \in [0,1]$ by

(2) \begin{equation*} w = 1 - r_R \end{equation*}

Intuitively, $w$ approaches 1 the more a gene set is ranked toward the top of the ranking. In the presence of ties, we calculate relative ranks

(3) \begin{equation*} r_R^* = P(S \ge s) \end{equation*}

as the fraction of gene sets with a value of the ranking statistic at least as extreme as observed for the gene set to be ranked [29]. Note that $r_R^* = r_R$ if there are no ties present in the ranking.

#### 2.6.2 Relevance score of an EA ranking

To assess the similarity of $R_{m(d)}$ with the corresponding relevance ranking $R_p$ for phenotype $p$, we compute the relevance score

(4) \begin{equation*} X_{m(d)} = \sum_{i=1}^{N_{GS}} w(i) S_p(i) ,\end{equation*}

where $w(i)$ is the weight of gene set $i$ in $R_{m(d)}$, and $S_p(i)$ is the relevance score of gene set $i$ in $R_p$. Intuitively, the greater the relevance score $S_p$ of a gene set, the more it is considered relevant for phenotype $p$. Also, the greater the relevance score $X_{m(d)}$ accumulated across the EA ranking, the more similar is the EA ranking $R_{m(d)}$ with the corresponding relevance ranking $R_p$. It can further be shown that the relevance score $X_{m(d)}$ has certain preferable properties over using a standard correlation measure or a standard classification performance measure such as the area under the ROC curve (Supplementary Methods S1.4).

#### 2.6.3 Empirical relevance score distribution

To assess whether the observed relevance score $X_{m(d)}$ significantly exceeds scores of a method placing the gene sets randomly along the ranking, we analogously compute relevance scores for random rankings of the gene sets and determine the proportion of random rankings achieving a score equal or greater than the observed score. To assess the significance of the observed relevance score while preserving ranking dependencies that are imposed by structural overlaps between gene sets, we also compute relevance scores for rankings obtained from the application of method $m$ to dataset $d$ with permuted sample labels and calculate the $P$-value as for a permutation test.

#### 2.6.4 Theoretical optimum

The observed relevance score $X_{m(d)}$ can be used to compare phenotype relevance of two or more EA methods when applied to one particular dataset. However, as the number of gene sets in the relevance rankings can differ between phenotypes, comparison between datasets is not straightforward as resulting relevance scores might scale differently (Supplementary Figures S12 and S13). Therefore, we compute the theoretically optimal score $O_p$ for the case $R_{m(d)} = R_p$ in which the EA ranking is identical to the relevance score ranking. The ratio

(5) \begin{equation*} \bar{X}_{m(d)} = X_{m(d)} / O_p \end{equation*}

between observed and optimal score can then be used when comparing scores obtained for several methods applied across multiple datasets. This allows one to assess whether certain EA methods tend to produce rankings of higher phenotype relevance than other methods when applied to a compendium of datasets.

### 2.7 Executable benchmark system

The GSEABenchmarkeR package is implemented in R [45] and is available from Bioconductor [46] under http://bioconductor.org/packages/GSEABenchmarkeR. The package allows one to (i) load specific pre-defined and user-defined data compendia, (ii) carry out DE analysis across datasets, (iii) apply EA methods to multiple datasets and (iv) benchmark results with respect to the chosen criteria. The individual components of the benchmark system are described in Supplementary Methods S1.5.

**Table 2:** Gene set analysis tools

**Tool**	**Author**	**Year**	**Citations** $^1$	**Availability**	**Gene sets**	**Methods** $^2$
WEBGESTALT	Zhang *et al*. [73]	2005	1423	Web server	GO, KEGG, +20 more	ORA, GSEA
GOSTATS	Falcon and Gentleman [74]	2007	1437	R package	GO	ORA
G:PROFILER	Reimand *et al*. [75]	2007	534	Web server	GO, KEGG, +7 more	ORA
GENETRAIL	Backes *et al*. [76]	2007	360	Web server	GO, KEGG, +28 more	ORA, GSEA
DAVID	Huang *et al*. [8]	2009	19 569	Web server	GO, KEGG, +38 more	ORA
GORILLA	Eden *et al*. [77]	2009	1881	Web server	GO	ORA
TOPPGENE	Chen *et al*. [78]	2009	1200	Web server	GO, KEGG, +45 more	ORA
CLUSTER-PROFILER	Yu *et al*. [10]	2012	1305	R package	GO, KEGG, +8 more	ORA, GSEA
PANTHER	Mi *et al*. [79]	2013	1405	Web server	GO, +2 more	ORA, GSEA
ENRICHR	Chen *et al*. [9]	2013	1246	Web server	GO, KEGG, +33 more	ORA

^1^Google Scholar, July 2019.

^2^Detailed summary of implemented methods in Supplementary Methods S1.2.

### 2.8 Research reproducibility

Results are reproducible using R and Bioconductor. Code is available from GitHub (https://github.com/waldronlab/GSEABenchmarking).

## 3 Results

We present the GSEABenchmarkeR R/Bioconductor package, which implements an executable benchmark framework for the systematic and reproducible assessment of gene set enrichment methods (Figure 1). The package facilitates efficient execution of a representative and extendable collection of EA methods on comprehensive experimental data compendia. The compendia are curated collections of microarray and RNA-seq datasets investigating human diseases (mostly specific cancer types), for which disease-relevant gene sets have been defined a priori. Consistently applied to these datasets, methods can then be assessed with respect to computational runtime, statistical significance and phenotype relevance, i.e. whether methods produce gene set rankings in which phenotype-relevant gene sets accumulate at the top. In the following, we use the package to assess the performance of 10 major EA methods listed in Table 1. These methods represent a decade of developments and are well established as indicated by their citation frequency.

### 3.1 Scope of the benchmark

We emphasize that the goal of our benchmark is a quantitative assessment of the performance of EA ‘methods/algorithms’ as opposed to a comparison of EA ‘tools’, typically facilitating the execution of one or more EA methods on a number of existing gene set databases with different options for result exploration and visualization (Table 2). We note that the methods in Table 1 are set-based and thus ignore known interactions between genes. We also note that benchmarking with the GSEABenchmarkeR package extends to network-based methods that incorporate known interactions (Supplementary Methods S1.6). However, as the assessment of network-based methods additionally requires evaluating the choice of network [14, 47], we decided to deal with these methods in a separate manuscript. As the universal inputs for all benchmarked methods, we consider (i) the full genes $\times $ samples expression matrix and (ii) a binary grouping vector that defines two sample groups in a case-control design, optionally supplemented by a blocking vector for paired samples or sample batches. We note that several methods also provide an execution mode that allows analysis of pre-ranked list of genes, which is useful for scenarios where the full expression matrix is not available, or where a gene list of interest has been derived from other genomic high-throughput assay types. On the other hand, we concentrate the benchmark on the majority of methods analyzing differences between sample groups. Enrichment methods scoring gene signature activities for single samples [15, 16], which can thus not be meaningfully compared to methods analyzing differences between sample groups, are not further considered. An exception is GSVA which applies single sample scoring of gene sets but can be used in conjunction with DE tools such as limma [38] to test for differences in gene set activity between sample groups. We start by exploring the benchmark compendia for sample size and DE and subsequently describe how EA methods developed for microarray data can be adapted for application to RNA-seq data.

### 3.2 Benchmark compendia and gene set collections

As illustrated in Figure 1, the two pre-defined benchmark compendia consist of 42 microarray datasets collected from GEO [48] by Tarca et al. [25, 26, GEO2KEGG] and 33 RNA-seq datasets from TCGA [32]. These datasets investigate 42 human diseases, including 35 cancer types (Supplementary Tables S1 and S2). When analyzing datasets of the benchmark compendia for sample size and DE, we find them to display a representative range (Supplementary Figure S1). The 42 datasets of the GEO2KEGG microarray compendium range from a minimum of 4 cases and 4 controls to a maximum of 91 cases and 62 controls. Using the typical DE thresholds of (i) absolute log2 fold change >1 and (ii) BH [41]-adjusted $P$-value < 0.05, we find several datasets of the GEO2KEGG microarray compendium with not a single DE gene, and at the other extreme, datasets with up to 73% DE genes (according to the $P$-value criterion; up to 15% satisfying both criteria). For this study, we restrict the analysis of the TCGA RNA-seq compendium to cancer types for which at least five adjacent normal tissue samples are available and take the pairing of samples (tumor vs. adjacent normal) into account. This yields 15 cancer types/datasets ranging from a minimum of 9 patients to a maximum of 226 patients, for which both tumor and adjacent normal samples were available (Supplementary Figure S1). Datasets of the TCGA RNA-seq compendium display relatively high levels of DE, with a range of 34–79% DE genes (according to the $P$-value criterion; 9–29% satisfying both criteria). We also explored the gene set size distribution in human KEGG pathways and GO-BP ontology (Supplementary Figure S2). When restricted to gene sets with a minimum of 5 genes and a maximum of 500 genes (the typical EA thresholds), we find that (i) there are considerable more GO-BP sets than KEGG sets (4631 vs. 323) and (ii) GO-BP gene sets tend to be smaller (median set size: 11 vs. 72).

### 3.3 Applicability to RNA-seq data

Popular methods for DE analysis require the raw RNA-seq read counts as input to preserve the sampling characteristics of the data [35, 39, 40]. However, frequently used tools for transcript abundance estimation report transcripts per million (TPMs) [49] or fragments/reads per kilobase of transcript per million mapped reads (FPKMs/RPKMs) [50] that already account for differences in gene length and sequencing depth. As FPKM/RPKM is inconsistent between samples and can be directly converted to TPM [49, 51], we consider raw read counts or TPMs as input for the EA methods under benchmark. Due to the different statistical models and implementations of the EA methods (Table 1), it is necessary to distinguish between (i) methods that work on the list of DE genes (ORA), which can be applied without modification assuming that gene length bias is controlled for [52], (ii) methods that distinguish between a microarray mode and an RNA-seq mode that assumes that the raw read counts are provided (CAMERA, ROAST and GSVA) or (iii) methods that incorporate sample permutation and recalculation of $t$-like statistics for each gene (GSEA, SAFE, GSA, SAMGS and PADOG). Methods of the third type require either a VST [37, 39] or incorporation of RNA-seq tools such as voom/limma, edgeR or DESeq2 for calculation of the per-gene statistic in each permutation [29, 53]. Incorporation of RNA-seq tools is straightforward for the permutation framework implemented in SAFE [54] as it allows one to provide user-defined local (per-gene) and global (gene set) test statistics. For the following assessment of EA methods, we thus also analyze the differences of using raw counts or VST-transformed counts as input. However, for the datasets of TCGA RNA-seq compendium, we observe almost identical fold changes and DE $P$-values when using either (i) voom/limma on raw read counts or TPMs or (ii) limma on VST-transformed counts or log TPMs (Supplementary Tables S4 and S5).

### 3.4 Runtime

**Figure 2:**
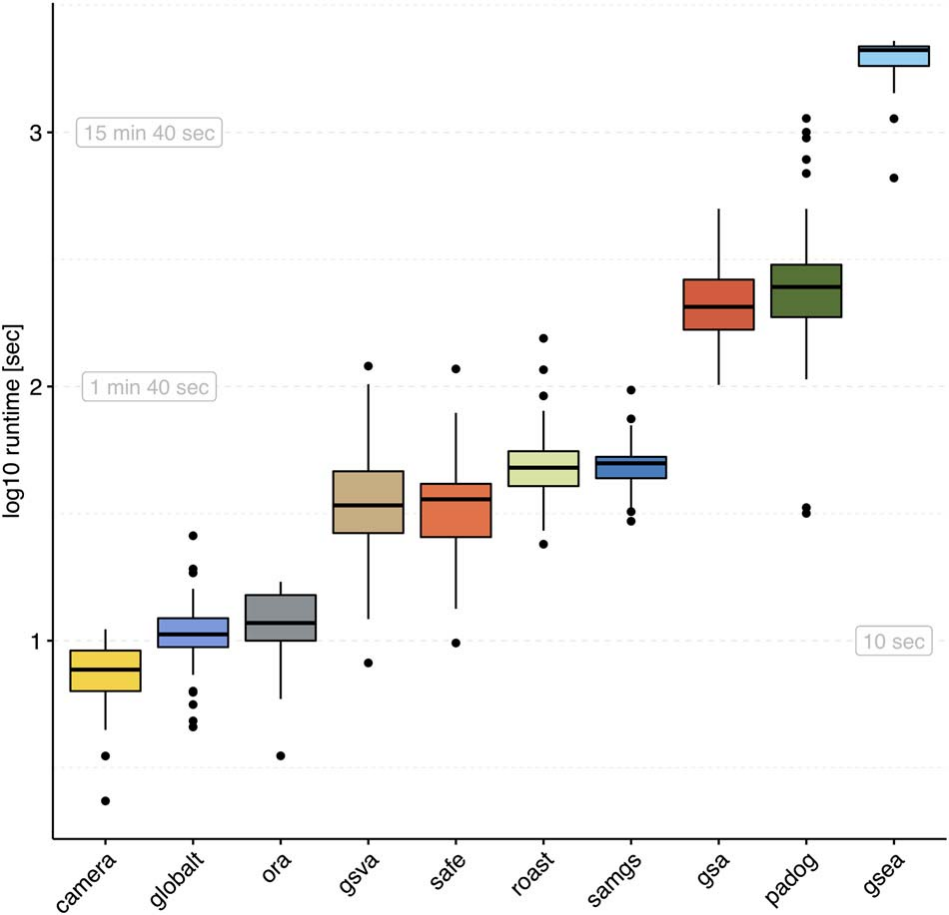
Runtime. Elapsed processing times ($y$-axis, log-scale) when applying the enrichment methods indicated on the $x$-axis to the 42 datasets of the GEO2KEGG microarray compendium. Gene sets were defined according to GO-BP ($N=4631$). Computation was carried out on an Intel Xeon 2.7 GHz machine. Runtimes for the TCGA RNA-seq compendium and when using KEGG gene sets are shown in Supplementary Figure S8.

The average per-dataset runtime in the microarray compendium using GO-BP gene sets (Figure 2) ranged from a minimum of 7.7 s (CAMERA) to a maximum of 32.6 min (GSEA). Closer inspection reveals three groups of methods reflecting aspects of methodology and implementation (Table 1). CAMERA, ORA and GLOBALTEST use simple parametric tests for gene set significance estimation, which results in fast runtimes. The other methods are computationally more intensive as they use sample permutation (SAFE, SAMGS, GSA, PADOG and GSEA) or Monte Carlo sampling (GSVA and ROAST). The most computationally expensive are GSA, PADOG and GSEA. Runtimes on the TCGA RNA-seq compendium and when using KEGG gene sets displayed a similar pattern (Supplementary Figure S3). However, we observed significantly increased runtimes when carrying out methods with dedicated RNA-seq mode on raw read counts (Supplementary Figure S4). This is especially apparent for the case of incorporating RNA-seq tools in the SAFE framework, where runtime also depends on which RNA-seq tool is used (voom/limma  $\ll $  edgeR  $\ll $  DESeq2).

### 3.5 Statistical significance

**Figure 3:**
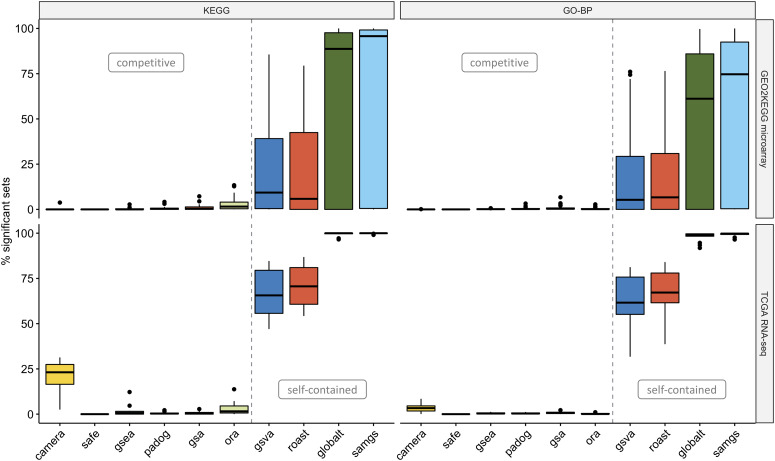
Statistical significance. Percentage of significant gene sets (FDR $< 0.05$, $y$-axis) when applying methods to the GEO2KEGG microarray compendium (top, 42 datasets) and the TCGA RNA-seq compendium (bottom, 15 datasets). Gene sets were defined according to KEGG (left, 323 gene sets) and GO-BP (right, 4631 gene sets). The gray dashed line divides methods based on the type of null hypothesis tested [6]. Supplementary Figure S8 shows the percentage of significant gene sets when using a nominal significance threshold of 0.05.

**Figure 4:**
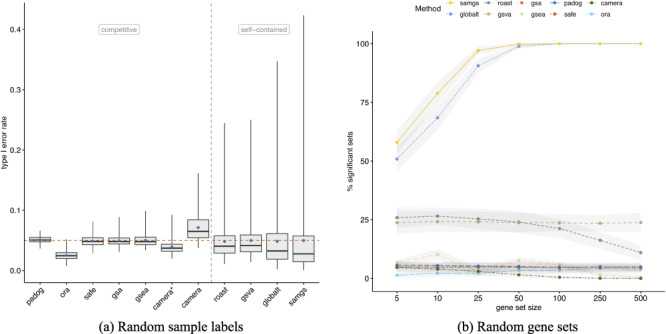
Random sample labels and random gene sets. **(a)** Type I error rates ($y$-axis) as evaluated on the dataset from Golub et al. [42] by shuffling sample labels 1000 times and assessing in each permutation the fraction of gene sets with $P < 0.05$. Gene sets were defined according to GO-BP ($N=4631$). Blue points indicate the mean type I error rate and the red dashed line the significance level of 0.05. The gray dashed line divides methods based on the type of null hypothesis tested [6]. *Application of CAMERA without accounting for inter-gene correlation (default: inter-gene correlation of 0.01). Supplementary Figure S5 shows type I error rates when using KEGG gene sets. Supplementary Figure S6 shows type I error rates for all four combinations of benchmark compendium and gene set collection. **(b)** Percentage of significant gene sets ($P < 0.05$, $y$-axis) when applying methods to the Golub dataset (true sample labels) and using 100 randomly sampled gene sets of defined size ($x$-axis). Shown is the mean $\pm $ standard deviation (gray bands) across 100 replications of the simulation experiment.

Enrichment methods conduct a hypothesis test for each gene set under investigation. The underlying null hypothesis can be characterized as either (i) self-contained: no genes in the set of interest are DE or (ii) competitive: the genes in the set of interest are at most as often DE as the genes not in the set [6]. As typically many gene sets are tested, multiple testing correction is needed to account for type I error rate inflation [55]. Using the popular BH method [41] for multiple testing correction and an adjusted significance level of 0.05, we find EA methods to report drastically different fractions of gene sets as statistically significant (Figure 3). This is tied to the type of null hypothesis tested, with self-contained methods reporting much larger fractions of significant gene sets. Conversely, we find several competitive methods (SAFE, GSEA, GSA and PADOG) to frequently report not a single significant gene set and two self-contained methods (GLOBALTEST and SAMGS) to frequently report all gene sets tested as significant. To ensure correct application of methods, we applied them in a controlled set-up (Figure 4). We therefore used the well-studied microarray dataset of [42] that contrasts the transcriptome profiles of AML and ALL patients. By shuffling sample labels (AML vs. ALL) 1000 times and assessing in each permutation the number of GO-BP gene sets with $P < 0.05$, we find average type I error rates controlled at the 5% level (Figure 4a). However, self-contained methods displayed in certain random assignments of the sample labels substantially elevated type I error rates. This effect was more pronounced for KEGG gene sets, which tend to be larger (Supplementary Figure S5). To test for a possible gene set size effect, we also applied methods to the Golub dataset (true sample labels) with randomly sampled gene sets of increasing size (Figure 4b). Self-contained methods reported systematically larger fractions of significant random gene sets, with GLOBALTEST and SAMGS displaying a set size dependency that resulted in rendering all random gene sets with >50 genes significant. This gene set size dependence was also apparent for both benchmark compendia, where self-contained methods reported larger fractions of significant gene sets for KEGG than for GO-BP (Figure 3). Following from the definition of the respective null hypothesis, self-contained but not competitive methods also display dependence on the background level of DE in a dataset (Supplementary Figure S7). As competitive methods were highly conservative, we inspected their nominal $P$-value distributions. Fraction of gene sets with nominal $P < 0.05$ were constant across datasets at $\approx $5–15% (Supplementary Figure S8), and the effect of the multiple testing correction was invariant to increasing the number of permutations or using the respective built-in FDR correction for GSEA and SAFE (Supplementary Figure S9).

### 3.6 Phenotype relevance

Evaluations of published EA methods often conclude phenotype relevance if there is any association between the top-ranked gene sets and the investigated phenotype. This involves a certain extent of cherry-picking from the enriched gene sets, where sets with a link to the phenotype are preferentially selected. For an impartial assessment, we propose to rather investigate phenotype relevance of all gene sets a priori and to subsequently quantify the relevance accumulated along the gene set ranking. For the non-trivial task of scoring the phenotype relevance of a gene set, we build on the MalaCards disease database [33]. MalaCards scores genes for disease relevance based on experimental evidence and co-citation in the literature and summarizes per-gene relevance across GO and KEGG gene sets (Supplementary Methods S1.3). Focusing on the diseases investigated in the datasets of the benchmark compendia, we systematically extracted disease genes and gene set relevance rankings from MalaCards (see again Figure 1). As expected, disease genes and gene sets for cancer types studied in the benchmark compendia (Supplementary Figures S11 and S12) are enriched for known cancer driver genes and oncogenic processes [56, 57]. Relevance rankings are also more similar within disease classes than between disease classes (Supplementary Figure S14).

**Figure 5:**
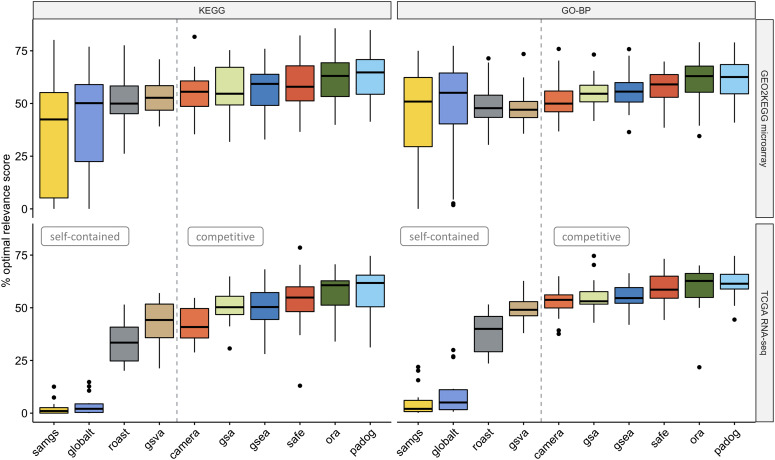
Phenotype relevance. Percentage of the optimal phenotype relevance score ($y$-axis) when applying methods to the GEO2KEGG microarray compendium (top, 42 datasets) and the TCGA RNA-seq compendium (bottom, 15 datasets). Gene sets were defined according to KEGG (left, 323 gene sets) and GO-BP (right, 4631 gene sets). The gray dashed line divides methods based on the type of null hypothesis tested [6]. The phenotype relevance score of a method $m$ applied to a dataset $d$ is the sum of the gene set relevance scores, weighted by the relative position of each gene set in the ranking of method $m$ (as outlined in Figure 1 and detailed in Phenotype relevance 2.6 section).

By scoring the similarity between the EA rankings and the precompiled relevance rankings, we assess whether certain EA methods tend to produce rankings of higher phenotype relevance (as outlined in Figure 1 and detailed in the Phenotype relevance 2.6 section). We observed that competitive methods tend to rank phenotype-relevant gene sets systematically higher than self-contained methods (Figure 5). This observation holds for all four combinations of benchmark compendium (GEO2KEGG and TCGA) and gene set collection (KEGG and GO-BP), resulting in a significant overall difference between competitive and self-contained methods ($P=1.87 \cdot 10^{-19}$, Wilcoxon rank-sum test). Differences between competitive methods were only moderate, with PADOG consistently returning highest relevance scores. However, PADOG scores were overall not significantly higher than ORA ($P=0.85$, Wilcoxon rank-sum test) and SAFE ($P=0.19$) but significantly exceeded the scores of GSEA ($P=0.014$), GSA ($P=0.04$) and CAMERA ($P=0.002$). We also confirmed that these observations largely hold when restricting the evaluation to the top 20% of each EA ranking (Supplementary Figure S15) and when inspecting accumulated relevance levels for individual datasets at varying thresholds of the MalaCards relevance score (Supplementary Figure S16).

## 4 Discussion

This article addresses two important gaps in the literature on GSEA. First, it implements a framework of software and data for rapid, comprehensive benchmarking of new or refined enrichment methods in a much larger and more diverse data compendium than used in previous benchmarking studies (Figure 1). Second, it applies this framework to benchmark 10 of the most widely used methods of EA for computational runtime (Figure 2), proportion of rejected null hypotheses (Figure 3), control of type I error rate (Figure 4) and biological relevance of gene set rankings (Figure 5). We distinguish between enrichment ‘methods’, which we benchmark here, and enrichment ‘tools’ which implement these methods. The enrichment methods benchmarked here are summarized in Table 1; popular enrichment tools are summarized in Table 2. We discuss enrichment methods in two broad categories: those employing a self-contained null hypothesis that no gene in the set is differentially expressed versus those employing a competitive null hypothesis that genes in the set are no more differentially expressed than genes outside the set [6]. This benchmarking provides the most comprehensive, data-based insight into the performance of gene set enrichment methods to date.

### Toward a gold standard for benchmarking GSEA

GSEA is among the most widely used approaches for interpreting transcriptomic experiments. Yet, these tools have been developed and published based on their performance in representative datasets that were based on microarray technology. Thus, there is a need for quantitative justification of the continued use of these methods, especially since the field has moved to RNA sequencing technology. This work curates a large and diverse benchmarking data compendium, including microarray and RNA-seq data, with a wide range of sample sizes and numerous outcome variables for differential expression analysis where some ground truth is known a priori. The KEGG and GO-BP gene set collections provide a range of biological processes and gene set size. Together, these datasets and gene sets provide an extensive testing ground for existing and new GSEA methods. The data are organized and presented through a well-documented Bioconductor [46] package GSEABenchmarkeR, which facilitates the analyses presented here as well as the plugging in of different benchmarks, enrichment methods, data and gene sets. By adopting GSEABenchmarkeR for standardized benchmarking, the field of GSEA can ensure that any newly proposed method provides a quantitative improvement over existing methods. Given its straightforward application to network-based methods, we anticipate that GSEABenchmarkeR will also greatly aid in resolving existing controversy concerning the effectiveness of network-based approaches when compared to set-based approaches, where evaluation of several choices of networks beyond pathway data in KEGG will be needed to arrive at a robust conclusion [14, 27, 31, 47].

### Applying enrichment methods to RNA-seq data

There is disagreement over whether, and how, enrichment methods originally developed for microarray data can be applied to RNA-seq data. This disagreement is amplified by the variety of RNA-seq expression units used at different steps of analysis. For instance, popular tools for differential expression analysis require the raw RNA-seq read counts as input to preserve the sampling characteristics of the data [35, 39, 40], whereas frequently used tools for transcript abundance estimation report TPMs [49] or FPKMs/RPKMs [50] that already account for differences in gene length and sequencing depth. We found that all enrichment methods developed for microarray data could be directly applied to RNA-seq data provided as raw read counts or TPMs through application of a VST and the same $t$-like gene-level statistics used for microarray data. These findings simplify the application of legacy enrichment methods to RNA-seq data and enable use of fast and established methods.

### Runtime

Runtime evaluation demonstrated moderate differences in applicability that mainly depend on methodological aspects and implementation. Consequently, we found simple parametric tests (CAMERA, ORA and GLOBALTEST) to complete a routine EA within seconds as compared to computationally more intensive permutation methods (GSA, PADOG and GSEA) that require several minutes. Although these runtimes are all within an acceptable range for typical use on a standard workstation, permutation-based methods may be inconvenient for larger gene set collections such as MSigDB [5]. Furthermore, the incorporation of differential expression methods for RNA-seq data such as edgeR or DESeq2 in permutation methods resulted in substantially increased runtimes without meaningfully altering results and is therefore not recommended.

### Statistical properties

The earliest enrichment methods continue to be the most frequently used, despite criticism of their statistical shortcomings. ORA (also sometimes referred to as Fisher’s Exact Test or Hypergeometric Test) is by far the most widely used enrichment method, employed by the most popular enrichment tools (Table 2). However, its use of the Hypergeometric Test assumes independence between the genes identified as differentially expressed, which is likely not the case [6, 58–61]. Furthermore, the permutation procedure incorporated in other widely used gene set tests has been shown to be biased [62] and inaccurate if permutation $P$-values are reported as zero [63]. Recent studies also reported non-uniform $P$-value distribution that is either systematically biased toward 0 (false positive inflation) or 1 (false negative inflation) [64, 65]. These shortcomings can lead to inappropriately small or large fractions of significant gene sets and can considerably impair prioritization of gene sets in practice. Our results demonstrate that the fraction of significant gene sets strongly depends on whether a self-contained or a competitive null hypothesis is tested. While the choice between a self-contained or a competitive method should be primarily motivated by the question at hand (testing for any association or testing for excess of differential expression in a gene set), it is important to keep in mind that this decision strongly influences which and how many gene sets are identified as enriched. Focusing on the practical implications of this analysis decision, we demonstrated that the choice can, in extreme cases, determine whether no gene sets (competitive) or all gene sets (self-contained) are identified as significantly enriched for the same dataset.

These dramatically different results require different approaches to interpretation and a trade-off when weighing type I versus type II error. For competitive methods, we found the fraction of significant gene sets to be constant across datasets at 5–15% using a nominal significance level of 0.05. When using competitive methods, it may thus be preferable to forego or relax multiple testing correction, especially when considering ranking and output of biologically plausible candidate gene sets for further exploration to be more important than a strict estimation of statistical significance. Such an approach is demonstrated by the interesting example of CAMERA, which deliberately abandons strict type I error control by default to compensate for the apparent lack in power of competitive methods [53, 66]. Self-contained methods, in contrast, tend to identify too many significant gene sets for significance to be a useful discriminating feature. Furthermore, with the exception of GSVA and ROAST, self-contained methods display gene set size dependency: even among random gene sets, larger gene sets are more likely identified as significant. We thus recommend GSVA or ROAST for analysts wanting a self-contained test. When stating significance of gene sets by a self-contained test, we recommend to also report the fraction of differentially expressed genes in the dataset, since this essentially determines the proportion of significant gene sets. However, we do note that it is not always straightforward to categorize methods as competitive or self-contained and that methods combining aspects from both models might either be predominantly or fully competitive or self-contained depending on the execution mode (Supplementary Discussion S2.1).

### Phenotype relevance

A critical objective of EA is to rank relevant gene sets higher than other gene sets. Quantitative benchmarking of this ability in experimental data, however, is difficult. We used aggregated relevance scores to determine whether certain methods tend to accumulate gene sets of high relevance toward the top of the ranking (for example, gene sets containing more known cancer-driving genes, established separately from the datasets used for benchmarking). This analysis demonstrated that competitive methods tend to rank relevant gene sets systematically higher than self-contained methods. PADOG consistently returned the highest relevance scores, which consolidates and extends previous assessments on microarray data that used a single target KEGG pathway per dataset [25, 26]. Although PADOG accumulated higher relevance scores than GSEA, we found ORA to provide equivalent relevance levels as PADOG. This underpins the usefulness of ORA as a fast and effective enrichment method, which might also explain its unbroken popularity [8, 67] despite methodological criticism [6, 58]. This is also in agreement with several previous assessments that demonstrated similar or better performance for conceptually simple enrichment methods [26, 27, 29, 59]. However, extrapolation to other ORA implementations than the one used here should be done with care, as results can differ depending on which genes are considered as differentially expressed and which genes are chosen as the background (Supplementary Discussion S2.2; [11, 66]). In the absence of a perfect gold standard with established ground truth, our evaluation of phenotype relevance generalizes human evaluation through biological reasoning based on associations reported in the literature. The evaluation thereby remains approximate, and further extension is warranted. This includes (i) replication of our findings on datasets not predominantly focusing on cancer types, (ii) to resolve cases where the relation between dataset and pre-defined relevant gene sets is not clear-cut and (iii) addressing limitations of the relevance rankings concerning their completeness and discriminatory power between related diseases (Supplementary Discussion S2.3). Such an extension to additional datasets and more fine-grained relevance rankings is straightforward in our benchmarking framework and will provide further important steps toward a gold standard for benchmarking of methods for GSEA.

## 5 Guidelines

For the exploratory analysis of **simple gene lists**, we recommend ORA given its ease of applicability, fast runtime and evident relevance of resulting gene set rankings, provided that input gene list and reference gene list are chosen carefully and remembering ORA’s propensity for type I error rate inflation when genes tend to be co-expressed within sets. For the analysis of **pre-ranked gene lists** accompanied by gene scores such as fold changes, alternatives to ORA such as pre-ranked GSEA [60] or pre-ranked CAMERA [66] exist (Supplementary Table S3). For expression-based EA on the **full expression matrix**, we recommend providing normalized log2 intensities for **microarray** data and logTPMs (or logRPKMs/logFPKMs) for **RNA-seq** data. When given raw read counts, we recommend to apply a VST such as voom [39] to arrive at library-size normalized logCPMs. If the question of interest is to test for association of any gene in the set with the phenotype (**self-contained** null hypothesis), we recommend ROAST or GSVA that both test a **directional** hypothesis (genes in the set tend to be either predominantly up- or down-regulated). Both methods can be applied for simple or extended experimental designs, where ROAST is the more natural choice for the comparison of sample groups and also allows one to test a **mixed** hypothesis (genes in the set tend to be differentially expressed, regardless of the direction). The main strength of GSVA lies in its capabilities for analyzing single samples. If the question of interest is to test for excess of differential expression in a gene set relative to genes outside the set (**competitive** null hypothesis), which we believe comes closest to the expectations and intuition of most end users when performing GSEA, we recommend PADOG, which is slower to run but resolves major shortcomings of ORA, and has desirable properties for the analyzed criteria and when compared to other competitive methods. However, PADOG is limited to testing a mixed hypothesis in a comparison of two sample groups, optionally including paired samples or sample batches. Therefore, we recommend the highly customizable SAFE for testing a directional hypothesis or in situations of more complex experimental designs such as comparisons between multiple groups, continuous phenotypes or the presence of covariates.

##  

**Key Points**

- The GSEABenchmarkeR R/Bioconductor package implements standards for reproducible benchmarking of enrichment methods.
- A VST of RNA-seq data unlocks enrichment methods originally developed for microarray data.
- The type of null hypothesis tested has strong implications for gene set testing in practice and can determine whether no gene sets or all gene sets are identified as enriched for the same dataset.
- Self-contained methods identify gene sets as enriched containing a single differentially expressed gene, a condition that is almost always true for larger gene sets and in datasets with higher levels of differential expression; ROAST and GSVA are recommendable for testing a self-contained null hypothesis.
- Competitive methods are more restrictive by testing for excess of differential expression in the gene set when compared to the background level, coming closer to the intuition of an enrichment and tend to rank relevant gene sets systematically higher than self-contained methods; ORA (simple gene list) and PADOG (full expression matrix) are recommendable for testing a competitive null hypothesis.

## Supplementary Material

supplement_bbz158

## Notes

**Ludwig Geistlinger** is a post-doctoral fellow in cancer genomics at the Graduate School of Public Health and Health Policy, City University of New York. His research focuses on methods for functional enrichment analysis of high-throughput genomic assay data. **Gergely Csaba** is a post-doctoral fellow at the Institute for Bioinformatics, Ludwig-Maximilians-Universität München. His research focuses on methods for gene expression data analysis, gene regulatory networks, protein structures and text mining. **Mara Santarelli** is a Master student at the Institute for Bioinformatics, Ludwig-Maximilians-Universität München. **Marcel Ramos** is a research associate at the Graduate School of Public Health and Health Policy, City University of New York. He is a member of the Bioconductor core team and develops integrative representations of multi-omics datasets. **Lucas Schiffer** is a PhD student in bioinformatics at Graduate School of Arts and Sciences, Boston University. His research focuses on statistical analysis of microbiome data. **Nitesh Turaga** is a software developer at the Department of Biostatistics and Bioinformatics, Roswell Park Comprehensive Cancer Center. He is a member of the Bioconductor core team and develops software for parallel computation on high-performance computer clusters. **Charity Law** is a post-doctoral fellow at the Epigenetics and Development Division of the Walter and Eliza Hall Institute of Medical Research. Her research focuses on statistical methods for RNA-seq analysis. **Sean Davis** is a senior associate scientist at the National Cancer Institute. He develops software for analyzing genomic data within the Bioconductor project. **Vincent Carey** is a professor of medicine at Harvard Medical School. He is a co-founder of the Bioconductor project with a focus on statistical analysis of genome-scale data. **Martin Morgan** is a professor of oncology at the Department of Biostatistics and Bioinformatics, Roswell Park Comprehensive Cancer Center. He leads the Bioconductor project with a focus on statistical methods in computational biology. **Ralf Zimmer** is a professor of bioinformatics at the Institute for Bioinformatics, Ludwig-Maximilians-Universität München. His research focuses on algorithms in bioinformatics with applications in gene expression data analysis, gene regulatory networks, protein structures and text mining. **Levi Waldron** is a professor of biostatistics at the Graduate School of Public Health and Health Policy, City University of New York. His research focuses on cancer genomics and metagenomic profiling of the human microbiome, where he develops methods within the intersection of statistical analysis and computation.
