# Supplementary material for: Toward a gold standard for benchmarking gene set enrichment analysis
Source: Brief Bioinform. 2020 Mar 9;22(1):545–56. doi: 10.1093/bib/bbz158 (PMC7820859; doi:10.1093/bib/bbz158)
Supplement: supplement_bbz158 [file supplement_bbz158.pdf]

Supporting Information

Contents

S1 Supplementary Methods **3**
S1.1 Enrichment methods . . . 3
S1.2 Enrichment tools . . . 5
S1.3 Construction of gene set relevance rankings from MalaCards . . . 6
S1.4 Comparison of the relevance score to alternative measures . . . 7
S1.4.1 Motivation and properties of the relevance score $X_{m(d)}$ . . . 7
S1.4.2 Alternative measures (true negative rate, ROC/AUC, cor, ...) . . . 7
S1.5 Executable benchmark system . . . 10
S1.6 Benchmarking network-based methods . . . 11
S1.7 Benchmarking user-defined methods . . . 12
S1.8 Incorporating user-defined benchmark compendia . . . 13
S1.9 Incorporating user-defined relevance rankings . . . 13

S2 Supplementary Discussion **14**
S2.1 Self-contained vs. competitive . . . 14
S2.2 ORA . . . 15
S2.2.1 Choosing the DE genes . . . 15
S2.2.2 Choosing the background . . . 15
S2.3 Limitations of the MalaCards relevance rankings . . . 16

List of Tables

S1 GEO2KEGG microarray compendium . . . 20
S2 TCGA disease codes . . . 21
S3 Gene set analysis methods under benchmark . . . 22
S4 Dealing with RNA-seq data: correlation of log2 fold changes . . . 22
S5 Dealing with RNA-seq data: correlation of -log10 DE p-values . . . 23

List of Figures

S1 Benchmark compendia: sample size and differential expression . . . 24
S2 Gene set size distribution . . . 25
S3 Runtime . . . 26
S4 Runtime for different RNA-seq modes . . . 27
S5 Random sample labels . . . 28
S6 Type I error rate . . . 29
S7 Correlation of differential expression and gene set enrichment . . . 30
S8 Statistical significance . . . 31
S9 Statistical significance with 10,000 permutations . . . 32
S10 Ranking granularity . . . 32
S11 MalaCards disease genes . . . 33
S12 MalaCards disease gene sets . . . 34
S13 MalaCards relevance score range . . . 35
S14 Similarity of MalaCards relevance rankings . . . 35

S15 Phenotype relevance (top 20%) . . . 36
S16 Accumulation of relevance score at different thresholds . . . 37
S17 Relevance score distribution per dataset (GEO2KEGG) . . . 38
S18 Relevance score distribution per dataset (TCGA) . . . 39

S1 Supplementary Methods

S1.1 Enrichment methods

In the following, we provide a brief overview of the main features of each enrichment method listed in Table 1 of the main manuscript with a focus on functionality, statistical approach, and implementation. Please refer to the references provided in Table 1 of the main manuscript for methodological details, and the R documentation of each method for implementation details. The categorization of each method as either self-contained or competitive is discussed in Supplementary Discussion S2.1.
All methods under benchmark were carried out through the `EnrichmentBrowser` package [1], which uses its own implementation of ORA, available `R`-scripts for GSEA and SAMGS, the corresponding `CRAN` package for GSA, and the respective `Bioconductor` packages for GLOBALTEST, SAFE, ROAST, CAMERA, PADOG, and GSVA.

ORA: Overrepresentation Analysis. Tests the overlap between the differentially expressed genes and genes in a gene set based on the hypergeometric distribution. Implementation: function `phyper` from the `stats` package. See Supplementary Discussion S2.1 for details.

GLOBALTEST: Global testing of groups of genes. Implements a self-contained test of groups of genes for association with a response variable. Implemented in the `globaltest` package [2]. We executed GLOBALTEST (`globaltest::gt`) providing the sample group vector (argument `response`), the expression dataset (`alternative`), the gene sets (`subsets`), and the number of permutations (`permutations`). Defaults were used for all other arguments. From the resulting `gt.object` we extracted the p-value for each gene set using the `p.value` accessor method.

GSEA: Gene Set Enrichment Analysis. Uses a Kolmogorov-Smirnov (KS) statistic to test whether the ranks of the p-values of genes in a gene set resemble a uniform distribution. Computation of the cumulative KS statistic can be time-consuming. Implementation: `R` script [3]. GSEA (function `GSEA` of the script) was executed providing the expression matrix (argument `input.ds`), the sample group vector (`input.cls`), the gene sets in GMT format (`gs.db`), and the number of permutations (`nperm`). Defaults were used for all other arguments. From the two tab-separated global result text files (one for gene sets with positive enrichment score, one for gene sets with negative enrichment score), we extracted the p-value for each gene set from the `NOM p-val` column.

SAFE: Significance Analysis of Function and Expression. Implements a general permutation framework that allows to combine values of a local (per-gene) test statistic within a global (gene set) test statistic. Uses Wilcoxon's rank sum as default global statistic. Implemented in the `safe` package [4]. SAFE (`safe::safe`) was executed providing the expression matrix (argument `X.mat`), the sample group vector (`y.vec`), the gene $\times$ gene set incidence matrix (`C.mat`), and the number of permutations (`Pi.mat`). Defaults were used for all other arguments, resulting in SAFE using Student's t as the gene level statistic (`local="t.Student"`) and Wilcoxon's rank sum as the gene set statistic (`global="Wilcoxon"`). From the resulting object we extracted the permutation p-value for each gene set from the `global.pval` slot.

GSA: Gene Set Analysis. Differs from GSEA by using the maxmean statistic, i.e. the maximum mean of either the positive or negative part of gene scores in the gene set. Uses a combination of sample permutation and gene permutation, which can result in longer runtimes. Implemented in the `GSA` package [5]. We executed GSA (`GSA::GSA`) providing the expression matrix (`x`), the sample group vector (`y`), the list of gene sets (`genesets`), and the number of permutations (`nperms`). Defaults were used for all other arguments, resulting in GSA using the maxmean statistic as method for computing the gene set statistic (`method="maxmean"`). From the result list we extracted the p-values for negative gene sets (`pvalues.lo`) and the p-values for positive gene sets (`pvalues.hi`), and calculated a single combined p-value for each gene set by taking the minimum p-value of the two directional p-values (positive / negative) multiplied

by two.

SAMGS: Significance Analysis of Microarrays on Gene Sets. Implements a self-contained test that extends the SAM method [6] for single genes to gene set analysis. Implementation: `R` script [7]. SAMGS (function `SAMGS` of the script) was executed providing the expression matrix (argument `DATA`), the sample group vector (`cl`), the gene $\times$ gene set incidence matrix (`GS`), and the number of permutations (`nbPermutations`). From the result we extracted the p-value for each gene set from the `GS p-value` column.

ROAST: ROtAtion gene Set Test. Implements a self-contained test that uses rotation instead of permutation for assessment of gene set significance. Implemented in the `limma` [8] and `edgeR` [9] packages for microarray and RNA-seq data, respectively. We executed ROAST (`limma::mroast`) providing the expression matrix (argument `y`), the gene set index list (`index`), the experimental design matrix (`design`), and the number of rotations (`nrot`). For raw RNA-seq read counts, the expression matrix was provided as a `DGEList` with size factors and dispersions calculated. Other arguments were left unchanged and carried out using the corresponding default value. From the result list we extracted the column `PValue`, containing the two-sided directional p-value for each gene set, which was used to generate the presented results.

CAMERA: Correlation Adjusted MEan RAnk gene set test. Implements a competitive test that accounts for inter-gene correlations. Implemented in the `limma` [8] and `edgeR` [9] packages for microarray and RNA-seq data, respectively. We executed CAMERA (`limma::camera`) providing the expression matrix (argument `y`), the gene set index list (`index`), and the experimental design matrix (`design`). For raw RNA-seq read counts, the expression matrix was provided as a `DGEList` with size factors and dispersions calculated. Other arguments were left unchanged and carried out using the corresponding default value. From the result list we extracted the column `PValue`, containing the two-tailed p-value for each gene set, which was used to generate the presented results.

PADOG: Pathway Analysis with Down-weighting of Overlapping Genes. PADOG computes the mean of the absolute moderated t-scores of the genes in a gene set, but additionally incorporates gene weights to favor genes appearing in few pathways versus genes that appear in many pathways. Implemented in the `PADOG` package [10]. PADOG (`PADOG::padog`) was executed providing the expression matrix (argument `esetm`), the sample group vector (`group`), the list of gene sets (`gslist`), and the number of permutations (`NI`). From the resulting `data.frame` we extracted the column `Ppadog`, containing the p-value for each gene set, which was used for all further analysis.

GSVA: Gene Set Variation Analysis. Transforms the data from a gene by sample matrix to a gene set by sample matrix, thereby allowing the evaluation of gene set enrichment for each sample. Implemented in the `GSVA` package [11]. We executed GSVA (`GSVA::gsva`) providing the expression matrix (argument `expr`), the list of gene sets (`gset.idx.list`), and `kcdf="Gaussian"` for continuous expression values (microarray log-intensities, RNA-seq log-CPMs or log-TPMs) and `kcdf="Poisson"` for raw RNA-seq read counts. Other arguments were left unchanged and carried out using the corresponding default value. Accordingly, GSVA were carried out with `mx.diff=TRUE` (default), calculating the enrichment score as the difference between the maximum positive and negative deviations from zero of the random walk statistic. To analyze differences in the enrichment scores between sample groups, we applied `limma` as suggested in the vignette of the `GSVA` package.

S1.2 Enrichment tools

The goal of our benchmark study is a quantitative assessment of the performance of EA *methods/algorithms* as opposed to a comparison of EA *tools*, typically facilitating the execution of one or more EA methods on a number of existing gene set databases with different options for result exploration and visualization. Popular enrichment tools listed in Table 2 of the main manuscript typically work on a list of genes provided by the user, and use a version of ORA and/or GSEA to quantify enrichment of genes annotated to specific molecular functions or biological process as e.g. defined in the Gene Ontology or the KEGG pathway database. In the following, we describe which methods each tool implements. Please refer to the references provided in Table 2 of the main manuscript for more information.

DAVID: uses ORA. Tests the overlap between the input gene list and genes in a gene set based on the hypergeometric distribution. The statistical test is identical to our implementation of ORA, but is often used with a much larger background set (the whole genome) which typically results in less conservative p-values (Supplementary Discussion S2.2.2).

GORILLA: provides two modes of hypergeometric testing. (1) standard ORA, identical to the one used by DAVID. (2) Application of ORA to all possible partitions of a ranked input list of genes and identification of the partition with minimum p-value.

GOSTATS: also provides two modes of hypergeometric testing. (1) standard ORA, but uses a non-specific filtering strategy to arrive at a more realistic background set (similar to the strategy described in Supplementary Discussion S2.2.2). (2) A conditional ORA that also takes into account the parent-child relationships between terms of the Gene Ontology.

WEBGESTALT: provides (1) standard ORA, identical to the one used by DAVID. (2) pre-ranked GSEA, which works on a ranked gene list instead of the full expression matrix [12]. (3) A network-based approach that performs random walk-based network propagation on an input gene list.

PANTHER: provides (1) standard ORA, identical to the one used by DAVID. (2) an enrichment test that is based on Wilcoxon's rank sum test, which is also used in SAFE per default.

CLUSTER-PROFILER: Similar to WEBGESTALT in providing (1) standard ORA, and (2) preranked GSEA.

ENRICHR: provides (1) standard ORA, identical to the one used by DAVID. (2) an empirical z-score method, that compares the observed rank for a gene set to the expected rank derived from performing ORA on many random input gene lists. (3) a combined score that multiplies the log of the ORA p-value from (1) with the z-score from (2).

TOPPGENE: uses standard ORA, identical to the one used by DAVID.

G:PROFILER: Similar to GORILLA in providing (1) standard ORA, and (2) application of ORA to all possible partitions of a ranked input list of genes and identification of the partition with minimum p-value.

GENETRAIL: supports enrichment analysis for (optionally ranked) input gene lists as the other tools above, but also implements methods for expression-based enrichment analysis when provided the full expression matrix (genes x samples). For simple gene lists, it provides standard ORA. For ranked gene lists that are optionally accompanied with scores it additionally provides pre-ranked GSEA and a number of additional set-level statistics. When provided a full expression matrix (such as an expression dataset from GEO), it allows similar to SAFE to choose from a number of local (gene-level) and global (set-level) statistics.

S1.3 Construction of gene set relevance rankings from MalaCards

As illustrated in Figure 1 of the main manuscript, we constructed gene set relevance rankings from `MalaCards` [13] in two steps. Focusing on the diseases investigated in the datasets of the benchmark compendia, we (1) systematically extracted relevant genes for each disease directly from the respective `MalaCards` page, and (2) subjected the disease genes for each disease to `GeneAnalytics` [14] to obtain the gene set relevance rankings for GO-BP terms and KEGG pathways. In the following we describe both steps in more detail. It is important to note that both steps of the curation process exclude any meaningful impact of (i) expression datasets of our benchmark compendium, and (ii) published gene set enrichment analyses of transcriptomic studies investigating diseases represented in our benchmark compendium.

1. Disease genes: According to `https://www.malacards.org/pages/info#related_genes`, the curation process of disease genes takes into account:

- `GeneCards` [15] textual association,
- Genetic testing resources supplying specific genetic tests for the disease,
- Genetic variations resources supplying specific causative variations in genes for the disease,
- Resources that manually curate the association of the disease with genes,

The sources for these evidence categories are (see Table 3 in [14]):

Evidence category	Source
Causative mutation	ClinVar, OMIM, Orphanet
Risk factor	ClinVar, OMIM, Orphanet
Resistant factor	ClinVar, OMIM
Genetic tests	GeneTests
Drug response	ClinVar
Structural gene variation	OMIM, Orphanet

According to `https://www.malacards.org/pages/info#scores` these evidence categories are combined into a composite score consisting of co-occurrences of gene and disease in `GeneCards` summaries, as well as the targeted experiments listed above, which do not include (1) high-throughput expression assays or (2) gene set enrichment analyses.
As an example, see the *Genes for Breast Cancer* section of `https://www.malacards.org/card/breast_cancer#related_genes`. For each gene it lists up to nine evidence categories including *Molecular basis known*, *Pathogenic*, *Causative variation*, *Genetic Tests*, and *Susceptibility factor*. One category is *GeneCards inferred* of which one sub-category is *Publications*. While this last category could include co-occurring text from the publications of our benchmark datasets, we note that our cancer vs. normal contrasts each generated from hundreds to thousands of differentially expressed genes. Even if a few of these genes were mentioned in the publications of the benchmark datasets, the impact on the MalaCards relevance score would be negligible.

2. Gene set relevance rankings: Given the list y of relevant genes for a disease D, `GeneAnalytics` [14] computes a composite relevance score $S(x)$ for a gene set x of interest (here: a GO-BP term or a KEGG pathway) via

$$S(x) = log_{10}\left(log_{100}(R(x))\prod_{g\in z} log_{100}(S_D(g))\right) + 10 + N_S \quad (1)$$

where $R(x)$ is the rank of gene set x, when ordering gene sets first by their `SetDistiller` [16] p-value, and then by gene set size. $S_D(g)$ is the disease relevance score of a gene g as derived in Step 1 above, and is taken into account for all genes in $z = x \cap y$, i.e. for genes that are shared between gene set x and the list y of relevant genes for disease D. N_S is the number of data sources supporting the disease relevance of genes in the gene set.
We observe that the gene set relevance score has three main components:

1. $R(x)$ is based on the `SetDistiller` [16] p-value, which is calculated from the binomial distribution, testing the null hypothesis that the frequency of gene set x in the query set y is not significantly different from what is expected with a random sampling of genes, given the frequency of the x in the set of all genes,
2. $\prod_{g \in z} S_D(g)$ summarizes the per-gene disease relevance for genes shared between the gene set x and the disease gene set y, and
3. N_S is the number of sources listed on a `GeneCards` page that support a link between genes in gene set x and disease D (see evidence categories and sources listed above).

We note that the `SetDistiller` test comes methodologically close to a hypergeometric ORA, but is carried out on the list of disease genes (as opposed to the list of DE genes for each dataset of the benchmark compendium). Further, it is used here as an additional weighting factor, where the main contribution to the gene set relevance score comes from the product of the per-gene disease relevance scores.

S1.4 Comparison of the relevance score to alternative measures

S1.4.1 Motivation and properties of the relevance score $X_{m(d)}$

In the manuscript we motivate the score as a measure of phenotype relevance accumulated along a gene set ranking. The score $X_{m(d)}$ for method m applied to dataset d therefore sums up the individual gene set relevance scores, weighted by the relative position of each gene set in the ranking of method m. Gene sets that are ranked towards the top obtain a high weight, and vice versa.
The goal of the score is to generalize the approach we take when manually inspecting a gene set ranking for phenotype relevance: we check whether top ranked gene sets ("positives") have relevance for the phenotype as reported in the literature ("true positives"). In general, we are thus interested in detecting a gene set *if it is relevant*. Researchers rarely inspect the bottom of the ranking / insignificant gene sets ("negatives"), and check whether these gene sets indeed have no relevance for the phenotype ("true negatives"). True negatives are also more difficult to establish as it can rarely be definitively determined whether a gene set is indeed irrelevant for a phenotype, or whether the gene set's relevance has simply not been studied or established yet (investigation bias / observational bias).
For additional properties and limitations of the relevance score $X_{m(d)}$ with respect to comparability between datasets and the presence of ties, we refer to Methods, Section 2.6 Phenotype relevance.

S1.4.2 Alternative measures (true negative rate, ROC/AUC, cor, ...)

It is instructive to inspect other measures. The `evalRelevance` function therefore accepts an argument `method` that determines how the relevance score is summarized across the enrichment analysis ranking (see the documentation of the function in the reference manual of the package). Choices for the `method` argument include:

- `"wsum"` to compute a weighted sum of the relevance scores (default, corresponds to $X_{m(d)}$);
- `"auc"` to perform a ROC/AUC analysis;

- "cor" to compute a correlation;
- or a user-defined function for customized behaviors.

Instead of "auc", this can also be any other performance measure that the ROCR package implements, for example "tnr" for calculation of the true negative rate.

However, the following considerations apply:

– **ROC / AUC / true negative rate**: It is tempting to treat the comparison of the EA ranking and the MalaCards ranking as a classification problem. However, this requires to divide (i) the EA ranking into enriched (positive) and not enriched (negative) gene sets, and (ii) the MalaCards ranking into relevant and irrelevant gene sets. Both steps are not straightforward and require the definition of thresholds. For (i), universal thresholding on the gene set p-value seems to lead to either overly small or large proportions of enriched gene sets depending on the method (Figure 3). And for (ii), the MalaCards ranking seem to rather provide varying degrees of relevance for a subset of gene sets, as opposed to a binary categorization as either relevant or irrelevant for all gene sets. Also, given the above considerations on the difficulty of establishing real true negatives, it is our understanding that absence from a MalaCards relevance ranking does not imply irrelevance for the phenotype *per se*. As a compromise, we consider a defined number of gene sets at the top of the EA ranking as enriched and, analogously, a defined number of gene sets at the top of the MalaCards ranking as relevant.

Let us consider the top 10 gene sets of the EA ranking as enriched and the top 10 gene sets of the MalaCards ranking as relevant. An optimal AUC of 1 is then achieved if all 10 relevant gene sets are placed (in arbitrary order) among the top 10 enriched gene sets. Note that such an analysis therefore does not allow to account for varying degrees of relevance among the 10 relevant gene sets: an optimal EA ranking placing the 10 relevant gene sets in the order of the relevance ranking achieves an AUC of 1; but also a suboptimal EA ranking that places the 10 relevant gene sets in reverse order at the top achieves an AUC of 1.

```
# setup
> library(GSEABenchmarkeR)

# MalaCards relevance ranking for Alzheimer's disease (KEGG pathways)
> rel.ranks
DataFrame with 57 rows and 2 columns
                                               TITLE REL.SCORE
                                         <character> <numeric>
hsa05010                          Alzheimers disease     84.12
hsa04932 Non-alcoholic fatty liver disease (NAFLD)     84.12
hsa04726                        Serotonergic synapse     49.19
hsa04728                        Dopaminergic synapse     49.19
hsa04713                       Circadian entrainment     49.19
...                                              ...       ...
hsa05310                                      Asthma      9.81
hsa05416                           Viral myocarditis      9.81
hsa05330                         Allograft rejection      9.81
hsa05332                   Graft-versus-host disease      9.81
hsa05321            Inflammatory bowel disease (IBD)      9.81

# an optimal EA ranking for which the top 10 enriched pathways ...
# ... correspond to the top 10 relevant pathways
> ea.ranks
DataFrame with 323 rows and 2 columns
                                                  GENE.SET      PVAL
                                               <character> <numeric>
1                              hsa05010_Alzheimers_disease  1.98e-12
2    hsa04932_Non-alcoholic_fatty_liver_disease_(NAFLD)      2.6e-11
```

```
3                          hsa04726_Serotonergic_synapse  3.43e-11
4                          hsa04728_Dopaminergic_synapse  1.28e-10
5                         hsa04713_Circadian_entrainment  7.08e-05
...                                                  ...       ...
319                                 hsa03040_Spliceosome     0.999
320   hsa04914_Progesterone-mediated_oocyte_maturation         1
321                          hsa00232_Caffeine_metabolism         1
322                  hsa00460_Cyanoamino_acid_metabolism         1
323        hsa00524_Butirosin_and_neomycin_biosynthesis         1

# AUC considering top 10 gene sets of relevance ranking as relevant (true positives)
> evalRelevance(ea.ranks, rel.ranks, method="auc", top=10)
[1] 1

# Now, place the 10 gene sets in reverse order at the top of the EA ranking ...
> ind <- c(10:1, 11:nrow(ea.ranks))
> ea.ranks.rev <- DataFrame(GENE.SET = ea.ranks$GENE.SET[ind], PVAL = ea.ranks$PVAL)
> ea.ranks.rev[1:10,]
DataFrame with 10 rows and 2 columns
                                            GENE.SET      PVAL
                                         <character> <numeric>
1                          hsa05211_Renal_cell_carcinoma  1.98e-12
2                          hsa04725_Cholinergic_synapse   2.6e-11
3       hsa04723_Retrograde_endocannabinoid_signaling  3.43e-11
4                        hsa04724_Glutamatergic_synapse  1.28e-10
5                            hsa04727_GABAergic_synapse  7.08e-05
6                        hsa04713_Circadian_entrainment  0.000118
7                          hsa04728_Dopaminergic_synapse   0.00174
8                          hsa04726_Serotonergic_synapse   0.00198
9   hsa04932_Non-alcoholic_fatty_liver_disease_(NAFLD)   0.00369
10                          hsa05010_Alzheimers_disease   0.00975

# ... and calculate AUC again
> evalRelevance(ea.ranks.rev, rel.ranks, method="auc", top=10)
[1] 1
```

This illustrates two beneficial aspects of the weighted sum $X_{m(d)}$: (1) it avoids any artificial thresholding on the EA ranking by calculating weights that express whether gene sets are rather ranked towards the top or the bottom of the ranking, and (2) it accounts for varying degrees of relevance in the MalaCards ranking. It thereby faithfully distinguishes between EA rankings that accumulate high phenotype relevance, but each ranking to a slightly different extent.

```
# optimal EA ranking
# (10 relevant pathways at the top, in the order of the relevance ranking)
> evalRelevance(ea.ranks, rel.ranks, method="wsum")
[1] 908.1592

# suboptimal EA ranking
# (10 relevant pathways at the top, but in reverse order)
> evalRelevance(ea.ranks.rev, rel.ranks, method="wsum")
[1] 905.8367
```

– **Correlation**: The main argument against using a standard correlation measure comes from the missingness in the relevance rankings (Supplementary Figure S12a and c). Thus, for a dataset and associated phenotype, we compare an EA ranking going over the full gene set vector ($N \approx 300$ for KEGG; $N \approx 4,600$ for GO-BP) against the typically much smaller vector of gene sets for which a relevance score is annotated. For this scenario, using rank correlation reduces the question to "does a *subset of the EA ranking* preserve the order of the relevance ranking"; although our question of interest is rather "is a *subset of the relevant gene sets* ranked highly in the EA ranking".

Consider the case of a relevance ranking containing 10 relevant gene sets against an EA ranking of 323 gene sets. Rank correlation (using `stats::cor` with `use = "pariwise.complete.obs"` and `method = "spearman"`) is then computed between the relevance ranking and the EA ranking restricted to the 10 relevant gene sets. This accordingly results in very different correlations for (i) an EA ranking that places the 10 relevant gene sets at the top (in the order of the relevance ranking), and (ii) an EA ranking that also places the 10 relevant gene sets at the top, but in reverse order.

```
# continuing with the relevance ranking for Alzheimer's disease from above, ...
# ... but restricting the relevance ranking to 10 pathways
> rel.ranks <- rel.ranks[1:10,]

# Spearman correlation for the optimal EA ranking
# (10 relevant pathways at the top, in the order of the relevance ranking)
> evalRelevance(ea.ranks,  rel.ranks, method="cor")
[1] 1

# Spearman correlation for the suboptimal EA ranking
# (10 relevant pathways at the top, but reverse order)
>  evalRelevance(ea.ranks.rev, rel.ranks, method="cor")
[1] -1
```

Here, the weighted sum $X_{m(d)}$ again has preferable properties, as it recognizes that both EA rankings accumulate high phenotype relevance at the top. Yet, as desired, a slightly higher score is obtained for the optimal ranking.

```
# Score of the optimal EA ranking
# (10 relevant pathways at the top, in the order of the relevance ranking)
> evalRelevance(ea.ranks, rel.ranks, method="wsum")
[1] 539.7688

# Score for the suboptimal EA ranking
# (10 relevant pathways at the top, but reverse order)
> evalRelevance(ea.ranks.rev, rel.ranks, method="wsum")
[1] 537.4463
```

S1.5 Executable benchmark system

The `GSEABenchmarkeR` package is implemented in `R` [17] and is available from `Bioconductor` [18] under `http://bioconductor.org/packages/GSEABenchmarkeR`. The package allows to (i) load specific pre-defined and user-defined data compendia, (ii) carry out DE analysis across datasets, (iii) apply EA methods to multiple datasets, and (iv) benchmark results with respect to the chosen criteria.

Loading of benchmark compendia is facilitated through the `loadEData` function, which simplifies access to (i) the pre-defined GEO2KEGG microarray compendium, (ii) the pre-defined TCGA RNA-seq compendium, and (iii) user-defined data from file. Datasets of the GEO2KEGG microarray compendium are loaded from the Bioconductor packages `KEGGdzPathwaysGEO` and `KEGGandMetacoreDzPathwaysGEO` [19, 20]. Probe-to-gene mapping for each dataset can optionally be carried out, in order to summarize expression levels for probes annotated to the same gene. Datasets of the TCGA RNA-seq compendium are loaded using the `curatedTCGAData` package [21] (TPMs) or from `GSE62944` [22, 23] (raw read counts). User-defined data is also accepted, requiring a file path to the directory where datasets have been saved as serialized `R` data files (Supplementary Methods S1.8).

Caching to flexibly save and restore an already processed expression data compendium is incorporated by building on functionality of the `BiocFileCache` package [24]. This is particularly beneficial as preparing an expression data compendium for benchmarking of EA methods can be time-consuming and can involve several pre-processing steps.

DE analysis between sample groups for selected datasets of a compendium can be carried out using the function `runDE`. The function invokes `deAna` on each dataset, which contrasts the sample groups depending on data type and user choice via `limma`/`voom`, `edgeR`, or `DESeq2`.

Enrichment analysis At the core of applying a specific EA method to a single dataset is the `runEA` function, which delegates execution of the chosen method to either `sbea` (set-based enrichment analysis) or `nbea` (network-based enrichment analysis). Both functions also accept user-defined enrichment methods (Supplementary Methods S1.7, [1]). In addition, `runEA` returns CPU time used and allows saving results for subsequent assessment.

Parallel computation of functions for microarray preprocessing, DE analysis, and enrichment analysis when applied to multiple datasets is realized by building on infrastructure implemented in the `BiocParallel` package [25]. Internally, these functions call `bplapply`, which per default triggers parallel computation as configured in `BiocParallel`'s registry of computation parameters. As a result, parallel computation is implicitly incorporated when calling these functions on a multi-core machine. To change the execution mode of these functions, accordingly configured computation parameters can either directly be registered, or supplied as an argument to the respective function. Distributed computation on an institutional computer cluster or a computing cloud is straightforward by similarly configuring a computation parameter of class `BatchtoolsParam` for that purpose.

Benchmarking Once methods have been applied to a chosen benchmark compendium, they can be subjected to a comparative assessment using dedicated functions for loading, evaluation, and visualization of the results. The function `evalNrSigSets` evaluates the fraction of significant gene sets given a significance level `alpha` and a method for multiple testing correction, which can be chosen from the methods implemented in `p.adjust` from the `stats` package. The function `evalRelevance` evaluates phenotype relevance between EA rankings and corresponding relevance rankings, given a mapping from dataset to phenotype investigated. Integrated relevance rankings can be refined and relevance rankings for additional datasets can also be incorporated (Supplementary Methods S1.9). Detailed documentation of all implemented functions is available in the reference manual of the package.

S1.6 Benchmarking network-based methods

Benchmarking with the `GSEABenchmarkeR` package extends to *network-based* methods that incorporate known gene regulatory interactions. For demonstration, we execute two network-based methods (SPIA [26] and GGEA [27]) on three datasets of the GEO2KEGG microarray compendium, and compare their runtimes on these datasets.

```
# setup
> library(GSEABenchmarkeR)
> library(EnrichmentBrowser)

# prepare
> geo2kegg <- loadEData("geo2kegg", nr.datasets=3, cache=FALSE)
> geo2kegg <- maPreproc(geo2kegg)
> geo2kegg <- runDE(geo2kegg)

# get KEGG gene sets
> kegg.gs <- getGenesets(org="hsa", db="kegg")

# compile a gene regulatory network from KEGG
> kegg.grn <- compileGRN(org="hsa", db="kegg")

# execute SPIA and GGEA on the three datasets
> res <- runEA(geo2kegg,
               methods=c("spia", "ggea"),
```

```
                 gs=kegg.gs,
                 grn=kegg.grn,
                 save2file=TRUE,
                 out.dir="~/nbea_bench")

# get the runtimes
> rtimes <- readResults(data.dir="~/nbea_bench", data.ids=names(geo2kegg),
                               methods=c("spia", "ggea"), type="runtime")
> rtimes
$spia
 GSE1297 GSE14762 GSE15471
 188.016  187.544  170.474

$ggea
 GSE1297 GSE14762 GSE15471
  58.351   45.705   46.612

# visualize comparative performance
> bpPlot(rtimes, what="runtime")

# pre-defined network-based methods
> EnrichmentBrowser::nbeaMethods()
[1] "ggea"        "spia"        "pathnet"     "degraph"     "ganpa"
[6] "cepa"        "topologygsa" "netgsa"
```

Listing 1: Benchmarking network-based methods

S1.7 Benchmarking user-defined methods

User-defined enrichment methods can easily be plugged into the benchmarking framework. For demonstration, we define a dummy enrichment method that randomly draws p-values from a uniform distribution. We then execute this method on datasets of the GEO2KEGG compendium and inspect the percentage of significant gene sets returned for each dataset.

```
# defining a new enrichment method
> method <- function(se, gs)
{
  ps <- runif(length(gs))
  names(ps) <- names(gs)
  return(ps)
}

# execute the method on the three datasets
> res <- runEA(geo2kegg,
                 methods=method,
                 gs=kegg.gs,
                 save2file=TRUE,
                 out.dir="~/method_bench")

# get the rankings
> ranks <- readResults(data.dir="~/method_bench", data.ids=names(geo2kegg),
                               methods="method", type="ranking")

# evaluate the percentage of significant gene sets
> sig.sets <- evalSigSets(ranks, padj="none", alpha=0.05)
> sig.sets
                     method
GSE1297            4.012346
GSE14762           3.076923
```

```
GSE15471            5.538462
```

Listing 2: Benchmarking user-defined methods

S1.8 Incorporating user-defined benchmark compendia

The benchmarking can be straightforward extended to additional datasets. The `loadEData` function accepts a directory where datasets of class `SummarizedExperiment` [28] are stored as `RDS` files [29].

```
# chosing a data directory from which additional datasets are loaded
> data.dir <- system.file("extdata", package="GSEABenchmarkeR")
> edat.dir <- file.path(data.dir, "myEData")

# loading from the chosen data directory
> edat <- loadEData(edat.dir)
> names(edat)
[1] "GSE42057x" "GSE7305x"

> edat[[1]]
class: SummarizedExperiment
dim: 50 136
metadata(5): experimentData annotation protocolData dataType dataId
assays(1): exprs
rownames(50): 3310 7318 ... 123036 117157
rowData names(0):
colnames(136): GSM1031553 GSM1031554 ... GSM1031683 GSM1031684
colData names(2): Sample GROUP
```

Listing 3: Incorporating user-defined benchmark compendia

S1.9 Incorporating user-defined relevance rankings

It is also possible to refine the integrated MalaCards relevance rankings or to incorporate relevance rankings for additional datasets. For demonstration, we define an exemplary relevance ranking for 10 gene sets, and evaluate the relevance accumulated by an exemplary EA ranking.

```
# (1) producing an EA ranking
> ea.ranks <- makeExampleData("ea.res")
> ea.ranks <- gsRanking(ea.ranks, signif.only=FALSE)
> ea.ranks
DataFrame with 10 rows and 2 columns
      GENE.SET      PVAL
   <character> <numeric>
1          gs3     0.007
2          gs4     0.009
3          gs9     0.037
4          gs7     0.039
5          gs6     0.041
6          gs5     0.351
7          gs8     0.437
8         gs10     0.558
9          gs1     0.835
10         gs2     0.978

# (2) defining a relevance score ranking
> rel.ranks <- ea.ranks
> rel.ranks[,2] <- round( runif(nrow(ea.ranks), min=1, max=100) )
> colnames(rel.ranks)[2] <- "REL.SCORE"
> rownames(rel.ranks) <- rel.ranks[,"GENE.SET"]
```

```
> ind <- order(rel.ranks[,"REL.SCORE"], decreasing=TRUE)
> rel.ranks <- rel.ranks[ind,]
> rel.ranks
DataFrame with 10 rows and 2 columns
        GENE.SET REL.SCORE
     <character> <numeric>
gs10        gs10        88
gs6          gs6        84
gs9          gs9        70
gs5          gs5        70
gs4          gs4        70
gs8          gs8        62
gs7          gs7        57
gs2          gs2        39
gs3          gs3        22
gs1          gs1        17

# (3a) evaluate relevance score
> evalRelevance(ea.ranks, rel.ranks)
[1] 266.9

# (3b) compute optimal score
> compOpt(rel.ranks, ea.ranks[,"GENE.SET"])
[1] 324.3

# (3c) relevance scores of random gene set rankings
> compRand(rel.ranks, ea.ranks[,"GENE.SET"], perm=3)
[1] 270.2 247.0 278.5
```

Listing 4: Incorporating user-defined relevance rankings

S2 Supplementary Discussion

S2.1 Self-contained vs. competitive

It is not always trivial to categorize methods as either competitive or self-contained, and several methods combine aspects from both models. For example, GSEA and SAFE are hybrid in the sense that they motivate their test statistic on the basis of a competitive gene-sampling model, but calculate their p-value in a self-contained subject-sampling manner [30]. This similarly applies for GSA, which computes a self-contained test statistic and calculate the p-value in a self-contained subject-sampling manner, but uses a competitive gene-sampling procedure for restandardization of the observed and permuted values of the test statistic, making GSA effectively competitive. PADOG also uses sample permutation and restandardization via gene permutation, but computes a mean of the absolute gene scores weighted by the occurrence frequency of genes across all gene sets tested. The classification of GSEA further depends on the execution mode: for small sample sizes, GSEA provides an argument to use gene permutation for the p-value calculation, making it fully competitive. When using sample permutation, GSEA can also be executed fully self-contained if the Kolmogorov-Smirnov statistic is calculated on the basis of the DE p-values for each gene in the gene set, instead of on their ranks [30].
Another interesting example is GSVA, that belongs to the class of single sample EA methods and thus takes a conceptually different approach than all other methods assessed in this paper. The other methods (1) analyze differential expression of individual genes between sample groups, and (2) summarize DE of individual genes across the gene set of investigation. Applied in a comparison of sample groups, GSVA reverses the typical approach by (1) computing gene set enrichment scores for each sample, and (2) testing for differential "expression" of these enrichment scores between sample groups using e.g. `limma`. While the second step is a self-contained significance assessment of the enrichment scores for each gene set, GSVA

computes the enrichment score for each sample like GSEA based on the KS-statistic in an "unsupervised" competitive way, i.e. without taking the sample classification into account [31]. Interestingly, we observed this approach to be effectively self-contained and closely resembling results obtained for ROAST, a fully self-contained method. We further note that such distinctions are not necessary for the competitive methods ORA and CAMERA, and the self-contained methods GLOBALTEST, SAMGS, and ROAST.

S2.2 ORA

S2.2.1 Choosing the DE genes

DE studies typically report a gene as differentially expressed if the corresponding DE p-value, corrected for multiple testing, satisfies the chosen significance level. EA methods that work directly on the list of DE genes are then substantially influenced not only by the DE method, but also by the method for multiple testing correction. ORA is inapplicable if there are few genes satisfying the significance threshold, or if almost all genes are DE. We therefore implemented a flexible, context-dependent adjustment procedure to account for such cases by applying multiple testing correction in dependence on the overall DE level in the dataset:

- the correction method from Benjamini and Hochberg (BH) is applied, if it renders $\geq 1\%$ and $\leq 25\%$ of all measured genes as DE,
- the p-values are left unadjusted, if the BH correction results in $< 1\%$ DE genes, and
- the more stringent Bonferroni correction is applied, if the BH correction results in $> 25\%$ DE genes.

Note that resulting p-values are not further used for assessing the statistical significance of DE genes within or between datasets. They are solely used to determine which genes are included in the analysis with ORA - where the context-dependent correction ensures that the fraction of included genes is roughly in the same order of magnitude across datasets.

S2.2.2 Choosing the background

Competitive gene set tests such as ORA compare the genes of the gene set tested against the background of genes not in the set [30]. Although rarely explicitly stated, the background is thus an important parameter [32], especially for the hypergeometric test used by ORA where it determines the size of the population from which genes are drawn [33]. We consider three different options for the population: (i) all genes measured in the microarray or RNA-seq experiment under study, (ii) all genes annotated in the gene set collection under study, and (iii) the intersection of (i) and (ii). While the differences between these three options seem subtle, the impact on the significance estimation of the hypergeometric test can be substantial.

We illustrate the impact of the choice of the background by considering a transcriptomic study, in which 12,671 genes have been tested for differential expression between two sample conditions and 529 genes were found DE. Among the DE genes, 28 are annotated to a specific gene set, which contains in total 170 genes. This setup corresponds to a 2 x 2 contingency table, where the overlap of 28 genes can be assessed based on the hypergeometric distribution. This corresponds to a one-sided version of Fisher's exact test, yielding here a highly significant enrichment.

```
> deTable <- matrix( c(28, 142, 501, 12000),
              nrow = 2,
              dimnames = list(c("DE", "Not.DE"),
                     c("In.gene.set", "Not.in.gene.set")))
> deTable
        In.gene.set Not.in.gene.set
```

```
DE                28              501
Not.DE           142            12000

> fisher.test(deTable, alternative = "greater")

  Fisher's Exact Test for Count Data

data:  deTable
p-value = 4.088e-10
alternative hypothesis: true odds ratio is greater than 1
95 percent confidence interval:
 3.226736       Inf
sample estimates:
odds ratio
  4.721744
```

Listing 5: Using all genes measured in the microarray or RNA-seq experiment under study

This setup would be realistic if all genes of the universe have equal chance to be drawn. However, due to overlaps between gene sets and missing annotation for other genes, some genes are preferentially drawn, and some genes cannot be drawn at all. To account for missing annotation, we restrict the population to genes annotated in the gene set collection under study. We illustrate this by using the human KEGG gene set collection that contains roughly 8,000 genes. The resulting p-value of the hypergeometric test drops by 4 orders of magnitude.

```
> kegg.gs <- EnrichmentBrowser::getGenesets(org="hsa", db="kegg")
> length(unique(unlist(kegg.gs)))
[1] 7852

> deTable[2,2] <- 8000
> fisher.test(deTable, alternative = "greater")

  Fisher's Exact Test for Count Data

data:  deTable
p-value = 1.207e-06
alternative hypothesis: true odds ratio is greater than 1
95 percent confidence interval:
 2.150785       Inf
sample estimates:
odds ratio
  3.147949
```

Listing 6: Using all genes annotated in the gene set collection under study

A similar argument can be made for genes in the gene set collection that are not measured, which is more common for microarray studies than for RNA-seq studies. To account for such genes, we restrict the population to the intersection of measured genes and annotated genes. For the example considered here, we assume the intersection to be 7,000 genes, reducing the p-value by another order of magnitude.

```
> deTable[2,2] <- 7000
> fisher.test(deTable, alternative = "greater")$p.value
[1] 1.240368e-05
```

Listing 7: Using the intersection of measured genes and annotated genes

S2.3 Limitations of the MalaCards relevance rankings

The MalaCards relevance rankings represent a systematic approach to quantifying phenotype relevance of gene sets based on experimental evidence and co-citation in the literature. However, there are three main limitations that leave room for future improvements:

- The MalaCards relevance rankings are incomplete. As the rankings are constructed from disease relevance of individual genes, only gene sets that contain at least one relevant gene are included in the rankings. However, in agreement with the considerations in Supplementary Methods S1.4 on investigation bias and observational bias, absence from a relevance ranking does not imply irrelevance for the phenotype *per se*. On the other hand, containing one or more relevant genes alone does not neccessarily imply relevance of the gene set as a whole, and a gene set's relevance might further depend on interplay between genes and context dependency of their activation.
- The relationship between dataset, investigated phenotype, and associated relevance ranking is not always clear-cut. Supplementary Figures S17 and S18 display the relevance score distribution of the overall high-scoring methods (the six competitive methods PADOG, ORA, SAFE, GSEA, GSA, CAMERA), identifying datasets of both benchmark compendia where those methods consistently return low scores. For those datasets further curational effort is required to clarify whether this inconsistency between the observed expression (in the dataset) and the expected expression (from the associated MalaCards relevance ranking) is due to (i) a not well-defined contrast between cases and controls, or (ii) a relevance ranking that is mainly based on experimental evidence types that are not detectable on the transcriptomic level.
- The relevance rankings have limited discriminatory power for related diseases. Supplementary Figure S14 demonstrates that the relevance rankings are partly very similar for different cancer types (especially the KEGG rankings). While this is expected to a certain extent given the universality of many cancer driver genes [34] and oncogenic pathways [35], supplementing the rankings with more fine-grained cancer type-specific information will likely also improve the accuracy of the phenotype relevance evaluation of the GSEA methods.

References

[1] Geistlinger, L., Csaba, G., Zimmer, R.: Bioconductor's EnrichmentBrowser: seamless navigation through combined results of set- & network-based enrichment analysis. BMC Bioinformatics **17**, 45 (2016)

[2] Barry, W.: GLOBALTEST Package. https://bioconductor.org/packages/globaltest

[3] GSEA Team: GSEA R Script. https://software.broadinstitute.org/cancer/software/gsea/wiki/index.php/R-GSEA_Readme

[4] Barry, W.: SAFE Package. https://bioconductor.org/packages/safe

[5] Efron, B., Tibshirani, R.: GSA Package. https://cran.r-project.org/package=GSA

[6] Tusher, V., Tibshirani, R., Chu, G.: Significance analysis of microarrays applied to the ionizing radiation response. Proc Natl Acad Sci U S A **98(9):**, 5116–21 (2001)

[7] Dinu, I: SAMGS R Script. http://www.ualberta.ca/yyasui/homepage.html

[8] Smyth, G.: Limma Package. https://bioconductor.org/packages/limma

[9] Robinson, M., McCarthy, D., Smyth, G.: edgeR Package. https://bioconductor.org/packages/edgeR

[10] Tarca, A.: PADOG Package. https://bioconductor.org/packages/PADOG

[11] Guinney, J.: GSVA Package. https://bioconductor.org/packages/GSVA

[12] Birger, C.: Pre-ranked GSEA. http://software.broadinstitute.org/cancer/software/genepattern/modules/docs/GSEAPreranked/1

[13] Rappaport, N., Twik, M., Nativ, N., *et al.*: MalaCards: A comprehensive automatically-mined database of human diseases. Curr Protoc Bioinformatics **47**, 1–24112419 (2014)

[14] Fuchs, S., Lieder, I., Stelzer, G., *et al.*: GeneAnalytics: an integrative gene set analysis tool for next generation sequencing, RNAseq and microarray data . OMICS **20(3)**, 139–51 (2016)

[15] Safran, M., Dalah, I., Alexander, J., *et al.*: Genecards version 3: the human gene integrator. Database **2010**, 020 (2010)

[16] Stelzer, G., Inger, A., Olender, T., *et al.*: GeneDecks: paralog hunting and gene-set distillation with GeneCards annotation. OMICS **13(6)**, 477–87 (2009)

[17] R Core Team: R: a Language and Environment for Statistical Computing. (2019). https://www.R-project.org

[18] Huber, W., Carey, V., Gentleman, R., *et al.*: Orchestrating high-throughput genomic analysis with Bioconductor. Nat Methods **12(2)**, 115–21 (2015)

[19] Tarca, A.L., Draghici, S., Bhatti, G., *et al.*: Down-weighting overlapping genes improves gene set analysis. BMC Bioinformatics **13**, 136 (2012)

[20] Tarca, A.L., Bhatti, G., Romero, R.: A comparison of gene set analysis methods in terms of sensitivity, prioritization and specificity. PLoS One **8(11)**, 79217 (2013)

[21] Ramos, M., Waldron, L., Schiffer, L., et al.: curatedTCGAData: curated data from The Cancer Genome Atlas (TCGA) as MultiAssayExperiment objects. doi:10.18129/B9.bioc.curatedTCGAData

[22] Rahman, M., Jackson, L.K., Johnson, W.E., *et al.*: Alternative preprocessing of RNA-sequencing data in The Cancer Genome Atlas leads to improved analysis results. Bioinformatics **31(22)**, 3666–72 (2015)

[23] Arora, S.: GEO accession data GSE62944 as a SummarizedExperiment. doi:10.18129/B9.bioc.GSE62944

[24] Shepherd, L., Morgan, M.: BiocFileCache: manage files across sessions. doi:10.18129/B9.bioc.BiocFileCache

[25] Morgan, M., Lawrence, M., Carey, V., et al.: BiocParallel: Bioconductor facilities for parallel evaluation. doi:10.18129/B9.bioc.BiocParallel

[26] Tarca, A.L., Draghici, S., Khatri, P., *et al.*: A novel signaling pathway impact analysis. Bioinformatics **25(1)**, 75–82 (2009)

[27] Geistlinger, L., Csaba, G., Küffner, R., Mulder, N., Zimmer, R.: From sets to graphs: towards a realistic enrichment analysis of transcriptomic systems. Bioinformatics **27(13)**, 366–73 (2011)

[28] Morgan, M., Obenchain, V., Hester, J., Pages, H.: SummarizedExperiment. doi:10.18129/B9.bioc.SummarizedExperiment

[29] R Core Team: Saving and Restoring R Data Files. https://www.rdocumentation.org/packages/base/versions/3.6.0/topics/readRDS

[30] Goeman, J.J., Bühlmann, P.: Analyzing gene expression data in terms of gene sets: methodological issues. Bioinformatics **23(8)**, 980–7 (2007)

[31] Rahmatallah, Y., Emmert-Streib, F., Glazko, G.: Gene set analysis approaches for RNA-seq data: performance evaluation and application guideline. Brief Bioinform **17(3)**, 393–407 (2016)

[32] Wu, D., Smyth, G.K.: Camera: a competitive gene set test accounting for inter-gene correlation. Nucleic Acids Res **40(17)**, 133 (2012)

[33] Huang, W., Sherman, B.T., Lempicki, R.A.: Bioinformatics enrichment tools: paths toward the comprehensive functional analysis of large gene lists. Nucleic Acids Res **37(1)**, 1–13 (2009)

[34] Bailey, M.H., Tokheim, C., Porta-Pardo, E., *et al.*: Comprehensive characterization of cancer driver genes and mutations. Cell **173(2)**, 371–85 (2018)

[35] Sanchez-Vega, F., Mina, M., Armenia, J., *et al.*: Oncogenic signaling pathways in The Cancer Genome Atlas. Cell **173(2)**, 321–37 (2018)

[36] Khatri, P., Sirota, M., Butte, A.J.: Ten years of pathway analysis: current approaches and outstanding challenges. PLoS Comput Biol **8(2)**, 1002375 (2012)

[37] Sergushichev, A.A.: An algorithm for fast preranked gene set enrichment analysis using cumulative statistic calculation. bioRxiv (2016). doi:10.1101/060012

[38] Birring, S., Pavord, I.: COPD: an autoimmune disease? Eur Respir J **38(2)**, 484 (2011)

Table S1: GEO2KEGG microarray compendium.

Dataset	Disease	Disease code
GSE14924_CD4	Acute myeloid leukemia	LAML
GSE14924_CD8	Acute myeloid leukemia	LAML
GSE9476	Acute myeloid leukemia	LAML
GSE1297	Alzheimer disease	ALZ
GSE16759	Alzheimer disease	ALZ
GSE5281_EC	Alzheimer disease	ALZ
GSE5281_HIP	Alzheimer disease	ALZ
GSE5281_VCX	Alzheimer disease	ALZ
GSE24739_G0	Chronic myeloid leukemia	CML
GSE24739_G1	Chronic myeloid leukemia	CML
GSE23878	Colorectal cancer	CRC
GSE4107	Colorectal cancer	CRC
GSE4183	Colorectal cancer	CRC
GSE8671	Colorectal cancer	CRC
GSE9348	Colorectal cancer	CRC
GSE19420	Diabetes mellitus type 2	DMND
GSE1145	Dilated cardiomyopathy	DCM
GSE3585	Dilated cardiomyopathy	DCM
GSE7305	Endometrial cancer	UCEC
GSE19728	Glioma	GBM
GSE21354	Glioma	LGG
GSE8762	Huntington disease	HUNT
GSE30153	Lupus erythematosus systemic	LES
GSE18842	Non small cell lung cancer	LUAD
GSE19188	Non small cell lung cancer	LUAD
GSE38666_epithelia	Ovarian neoplasms	OV
GSE38666_stroma	Ovarian neoplasms	OV
GSE15471	Pancreatic cancer	PAAD
GSE16515	Pancreatic cancer	PAAD
GSE22780	Pancreatic neoplasms	PAAD
GSE32676	Pancreatic cancer	PAAD
GSE20153	Parkinson disease	PARK
GSE20164	Parkinson disease	PARK
GSE20291	Parkinson disease	PARK
GSE6956AA	Prostate cancer	PRAD
GSE6956C	Prostate cancer	PRAD
GSE11906	Pulmonary disease chronic obstructive	PDCO
GSE42057	Pulmonary disease chronic obstructive	PDCO
GSE14762	Renal cancer	KIRC
GSE781	Renal cancer	KIRC
GSE3467	Thyroid cancer	THCA
GSE3678	Thyroid cancer	THCA

Table S2: TCGA disease codes.

Disease code	Disease
ACC	Adrenocortical carcinoma
BLCA	Bladder Urothelial Carcinoma
BRCA	Breast invasive carcinoma
CESC	Cervical squamous cell carcinoma and endocervical adenocarcinoma
CHOL	Cholangiocarcinoma
COAD	Colon adenocarcinoma
DLBC	Lymphoid Neoplasm Diffuse Large B-cell Lymphoma
ESCA	Esophageal carcinoma
GBM	Glioblastoma multiforme
HNSC	Head and Neck squamous cell carcinoma
KICH	Kidney Chromophobe
KIRC	Kidney renal clear cell carcinoma
KIRP	Kidney renal papillary cell carcinoma
LAML	Acute Myeloid Leukemia
LGG	Brain Lower Grade Glioma
LIHC	Liver hepatocellular carcinoma
LUAD	Lung adenocarcinoma
LUSC	Lung squamous cell carcinoma
MESO	Mesothelioma
OV	Ovarian serous cystadenocarcinoma
PAAD	Pancreatic adenocarcinoma
PCPG	Pheochromocytoma and Paraganglioma
PRAD	Prostate adenocarcinoma
READ	Rectum adenocarcinoma
SARC	Sarcoma
SKCM	Skin Cutaneous Melanoma
STAD	Stomach adenocarcinoma
TGCT	Testicular Germ Cell Tumors
THCA	Thyroid carcinoma
THYM	Thymoma
UCEC	Uterine Corpus Endometrial Carcinoma
UCS	Uterine Carcinosarcoma
UVM	Uveal Melanoma

Table S3: Gene set analysis methods under benchmark (continued).

Method	Null hypothesis[1]	Generation[2]	Directionality[3]	Pre-ranked[4]	Experimental design
ORA	Competitive	Overrepresentation	Mixed (default)	Available	Arbitrary
GLOBALTEST	Self-contained	–	Mixed (default)	Not available	Design matrix
GSEA	Competitive	Gene set scoring	Directional	Available	Two groups
SAFE	Competitive	Gene set scoring	Directional (default)	Not available	Various
GSA	Competitive	Gene set scoring	Directional	Not available	Various
SAMGS	Self-contained	Gene set scoring	Mixed	Not available	Two groups
ROAST	Self-contained	Gene set scoring	Directional (default)	Not available	Design matrix
CAMERA	Competitive	Gene set scoring	Directional	Available	Design matrix
PADOG	Competitive	Gene set scoring	Mixed	Not available	Two groups
GSVA	Self-contained	–	Directional	Not available	Single sample

[1] See Supplementary Discussion S2.1 for details

[2] Generations as defined by Khatri *et al.* [36] and in the Introduction section of the main manuscript.

[3] A *directional* method tests whether genes in the set tend to be either predominantly up- or down-regulated; a *mixed* method tests whether genes in the set tend to be differentially expressed, regardless of the direction.

[4] Analysis of pre-ranked list of genes, which is useful for scenarios where the full expression matrix is not available. ORA: choose from the tools listed in Table 2 of the main manuscript. GSEA: popular implementations include `GSEAPreranked` [12] and `fgsea` [37]. CAMERA: part of the `limma` [8] package.

Table S4: Dealing with RNA-seq data: correlation of log2 fold changes. Using the log2 fold changes obtained from applying `voom`/`limma` to the raw read counts available from GSE62944 as a reference, the table shows Pearson correlation with log2 fold changes obtained from applying `limma` subsequent to a variance stabilizing transformation (VST) on the raw read counts (2nd column), or `voom`/`limma` on TPMs available from `curatedTCGAData` (3rd column), or `limma` on the log2-transformed TPMs.

Dataset	VST + limma	TPM + voom/limma	log2 TPM + limma
BLCA	0.971	0.993	0.966
BRCA	0.991	0.996	0.987
COAD	0.995	0.979	0.978
HNSC	0.982	0.998	0.982
KICH	0.99	0.996	0.983
KIRC	0.992	0.997	0.988
KIRP	0.979	0.995	0.967
LIHC	0.964	0.995	0.956
LUAD	0.993	0.993	0.985
LUSC	0.993	0.997	0.989
PRAD	0.991	0.992	0.985
READ	0.992	0.987	0.984
STAD	0.975	0.992	0.967
THCA	0.987	0.994	0.98
UCEC	0.985	0.992	0.978

Table S5: Dealing with RNA-seq data: correlation of -log10 DE *p*-values. See caption of Table S4.

Dataset	VST + limma	TPM + voom/limma	log2 TPM + limma
BLCA	0.976	0.98	0.963
BRCA	0.987	0.986	0.96
COAD	0.989	0.954	0.921
HNSC	0.983	0.992	0.978
KICH	0.976	0.986	0.875
KIRC	0.985	0.989	0.964
KIRP	0.964	0.975	0.924
LIHC	0.97	0.985	0.964
LUAD	0.989	0.971	0.963
LUSC	0.986	0.992	0.975
PRAD	0.992	0.959	0.948
READ	0.984	0.97	0.964
STAD	0.982	0.978	0.951
THCA	0.979	0.984	0.929
UCEC	0.98	0.976	0.965

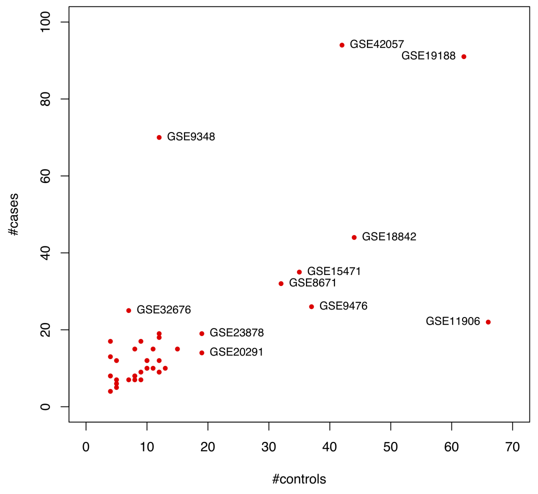

(a) GEO2KEGG: number of samples

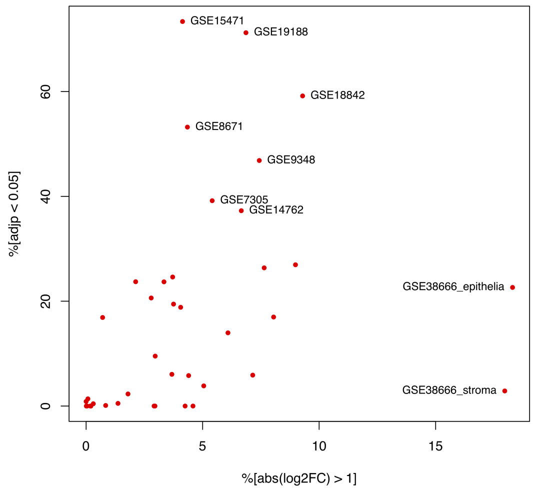

(b) GEO2KEGG: percentage of DE genes

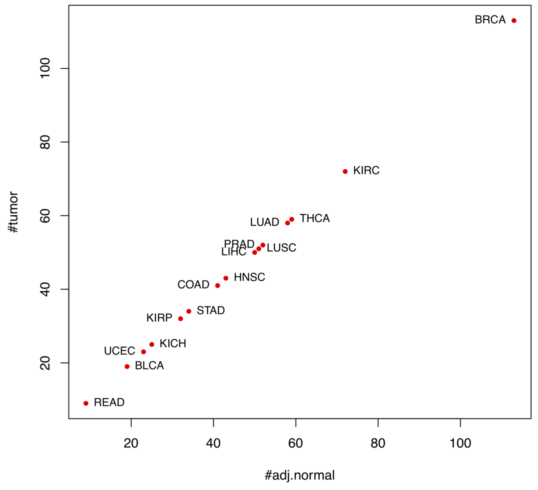

(c) TCGA: number of samples

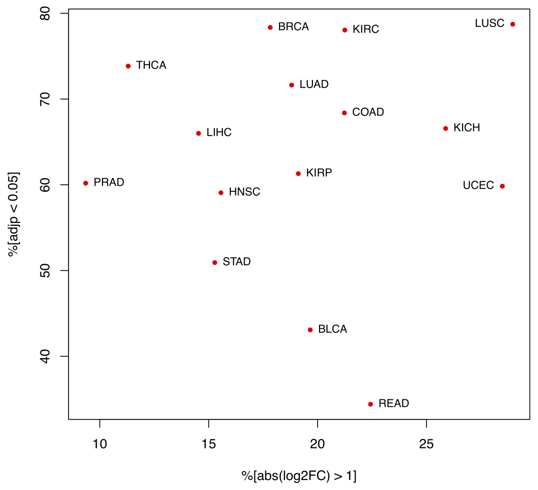

(d) TCGA: percentage of DE genes

Figure S1: Benchmark compendia: sample size and differential expression. Panel **(a)** and **(c)** show the number of cases and controls for each dataset of the GEO2KEGG microarray compendium ($N = 42$) and the TCGA RNA-seq compendium ($N = 15$), respectively. Using the typical thresholds for differential expression (DE), panel **(b)** and **(d)** show the percentage of genes with an absolute log2 fold change above 1 (x-axis) and a Benjamini-Hochberg (BH)-adjusted p-value below 0.05 (y-axis).

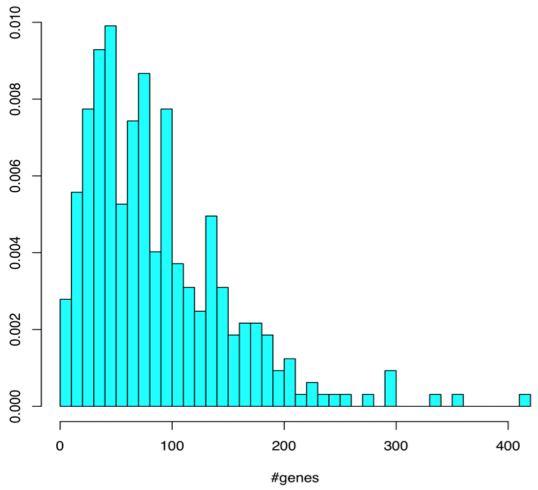

(a) KEGG

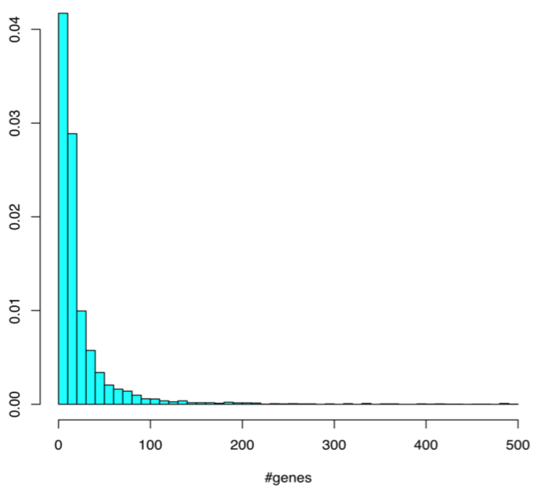

(b) GO-BP

Figure S2: Gene set size distribution. Considering only gene sets with a minimum of 5 genes and a maximum of 500 genes (the typical thresholds for EA analysis), gene set size distributions are shown for **(a)** 323 human KEGG gene sets (median set size of 72 genes), and **(b)** 4,631 human GO-BP gene sets (median set size of 11 genes). Filtering for set size was applied on a total of 331 KEGG gene sets and 12,078 GO-BP gene sets.

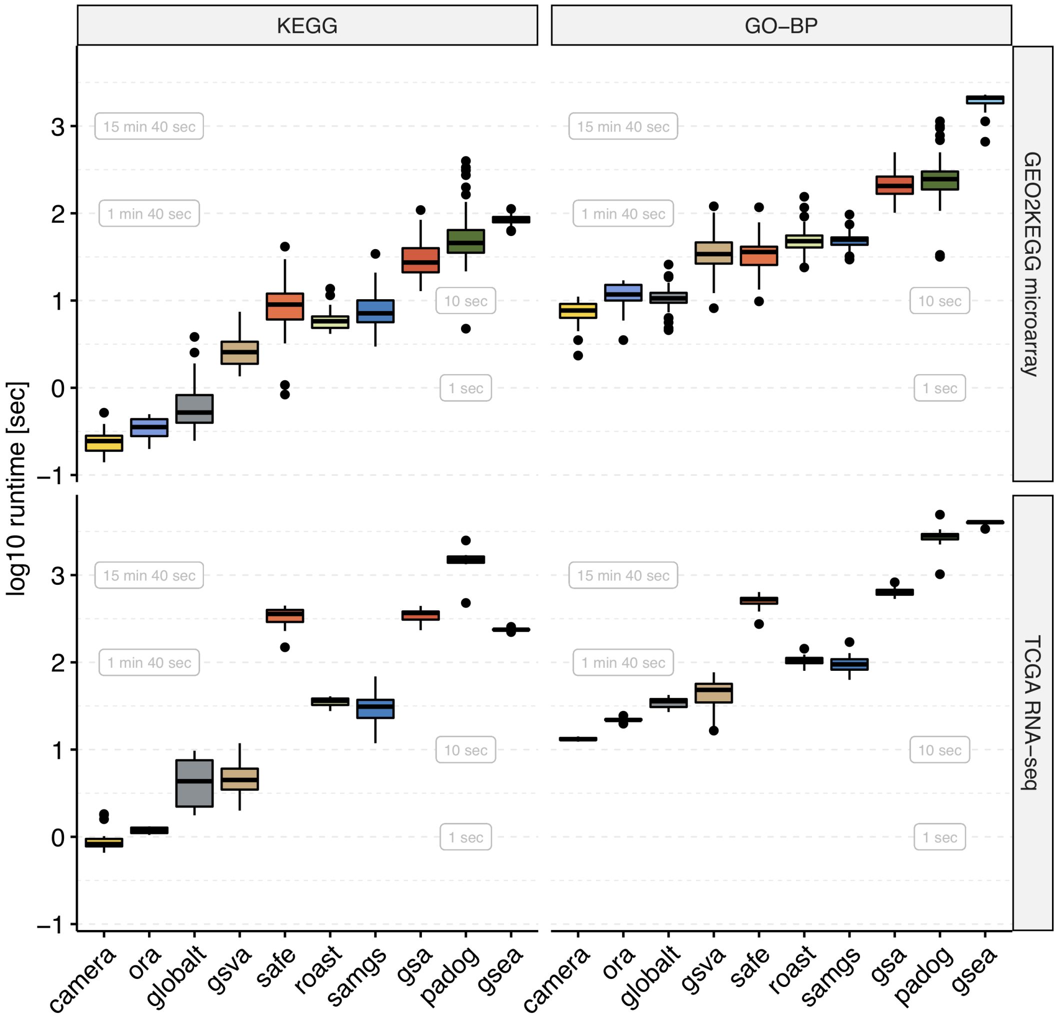

Figure S3: Runtime. Shown are the distributions of the elapsed processing times (y-axis, log-scale) when applying the enrichment methods indicated on the x-axis to the GEO2KEGG microarray compendium (top, 42 datasets) and the TCGA RNA-seq compendium (bottom, 15 datasets). Gene sets were defined according to KEGG (left, 323 gene sets) and GO-BP (right, 4,631 gene sets). Computation was carried out on an Intel Xeon 2.7 GHz machine.

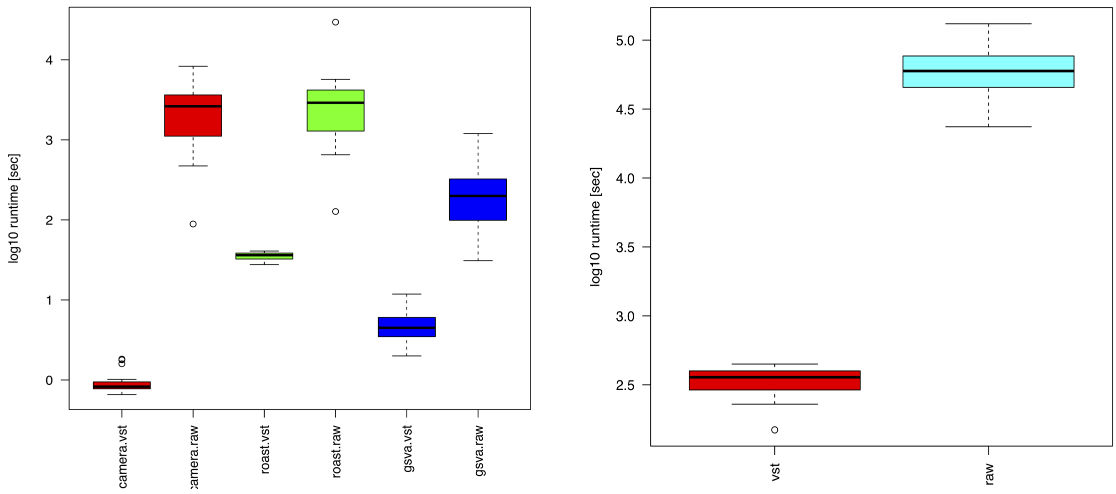

(a) CAMERA, ROAST, GSVA **(b)** SAFE

Figure S4: Runtime for different RNA-seq modes. Shown are the distributions of the elapsed processing times (y-axis, log-scale) when applying the enrichment methods indicated on the x-axis to the TCGA RNA-seq compendium (15 datasets). Gene sets were defined according to KEGG (323 gene sets). Computation was carried out on an Intel Xeon 2.7 GHz machine. VST: application of methods in microarray mode after applying a variance stabilizing transformation (VST) to the raw RNA-seq read counts. RAW: application of methods in RNA-seq mode to the raw RNA-seq read counts. For SAFE, application to the raw RNA-seq read counts was carried out by using `voom`/`limma` for recalculation of the local (per-gene) statistic in each permutation of the sample labels.

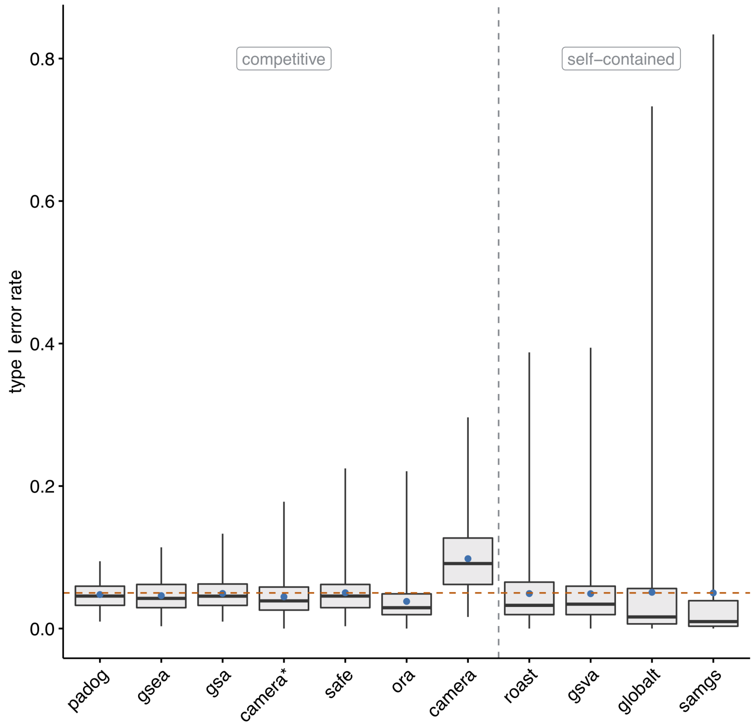

Figure S5: Random sample labels. Type I error rates (y-axis) as evaluated on the Golub dataset by shuffling sample labels 1000 times, and assessing in each permutation the fraction of gene sets with $p < 0.05$. Gene sets were defined according to KEGG ($N = 323$). Blue points indicate the mean type I error rate. The red dashed line indicates the significance level of 0.05. The grey dashed line divides methods based on the type of null hypothesis tested.

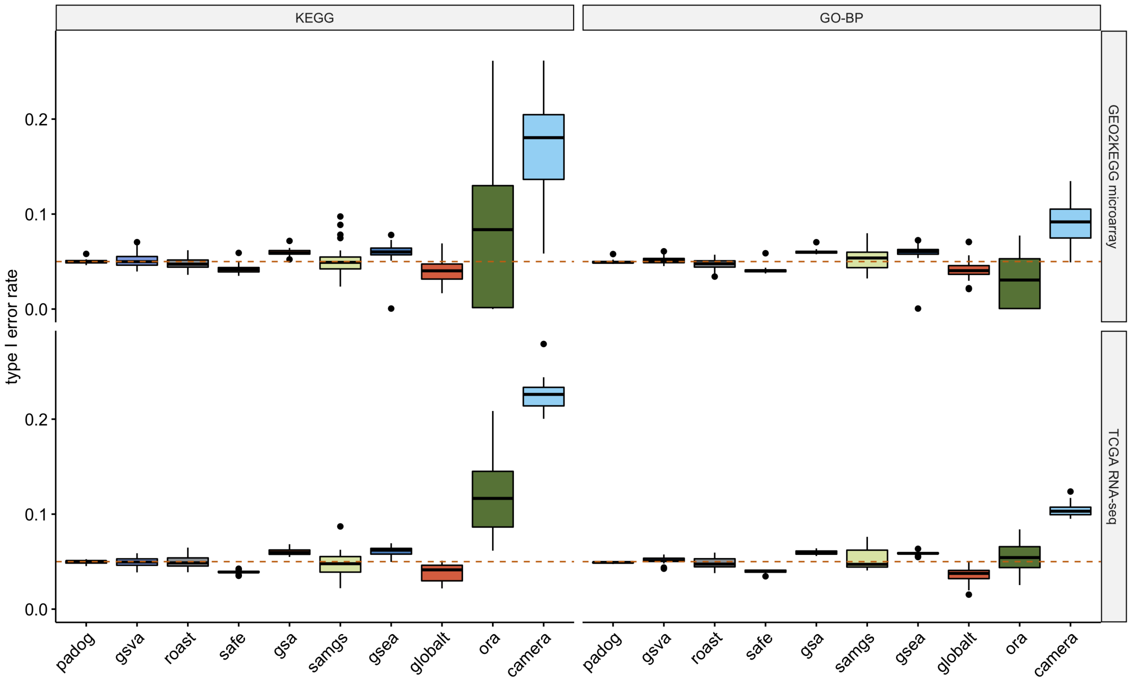

Figure S6: Type I error rate. Mean type I error rates (y-axis) when applying methods to the GEO2KEGG microarray compendium (top, 42 datasets) and the TCGA RNA-seq compendium (bottom, 15 datasets). Gene sets were defined according to KEGG (left, 323 gene sets) and GO-BP (right, 4,631 gene sets). Type I error rates were computed for each dataset of the benchmark compendia by sample label permutation as described for the Golub dataset in Supplementary Figure S5. Note that we show for each method the distribution of the *mean* type I error rate per dataset (blue points in Supplementary Figure S5).

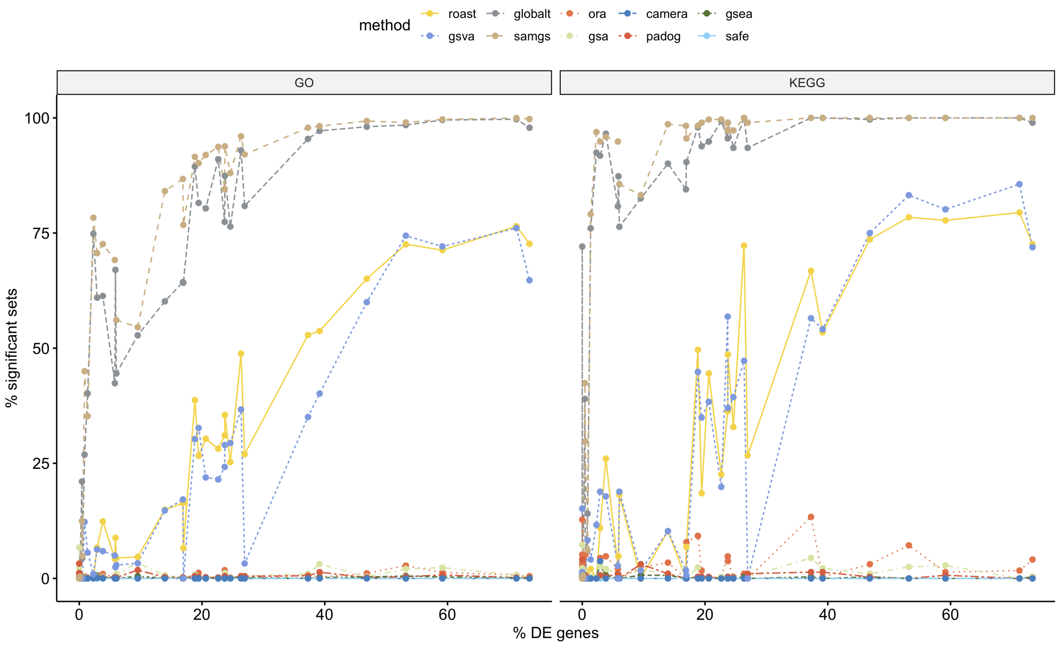

Figure S7: Correlation of differential expression and gene set enrichment. Percentage of significant gene sets (FDR < 0.05, y-axis) when applying methods to the GEO2KEGG microarray compendium (42 datasets) as a function of the percentage of DE genes (FDR < 0.05, x-axis). Gene sets were defined according to GO-BP (left panel) and KEGG (right panel). The plots show strong correlation for the self-contained methods ROAST (Pearson correlation of 0.968 for GO, and 0.91 for KEGG), GSVA (0.947, 0.903), GLOBALTEST (0.792, 0.637), and SAMGS (0.745, 0.631).

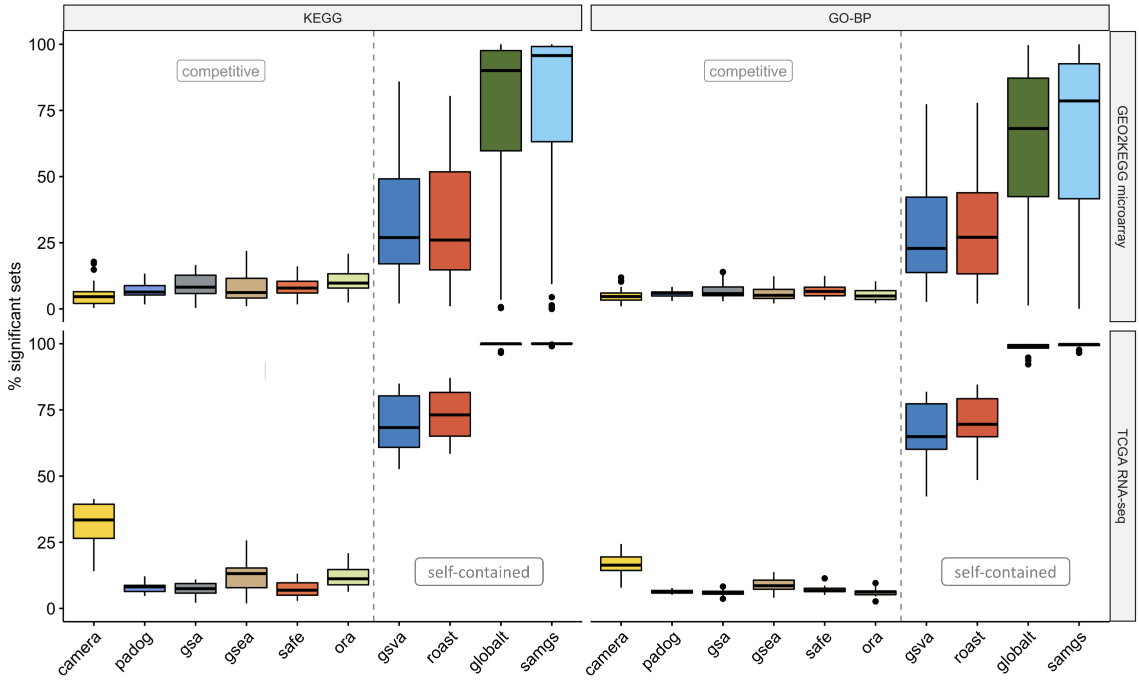

Figure S8: Statistical significance. Percentage of significant gene sets before multiple testing correction ($p < 0.05$, y-axis) when applying methods to the GEO2KEGG microarray compendium (top, 42 datasets) and the TCGA RNA-seq compendium (bottom, 15 datasets). Gene sets were defined according to KEGG (left, 323 gene sets) and GO-BP (right, 4,631 gene sets). The grey dashed line divides methods based on the type of null hypothesis tested.

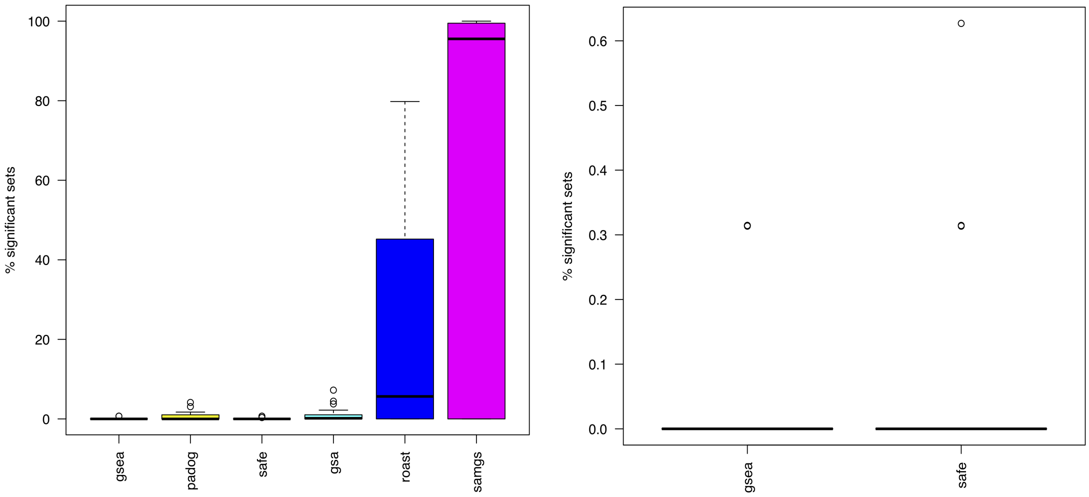

(a) p.adjust=BH **(b)** built-in FDR

Figure S9: Statistical significance with 10,000 permutations. Percentage of significant gene sets (FDR < 0.05, y-axis) when applying methods with 10,000 permutations to the GEO2KEGG microarray compendium (42 datasets) and using KEGG gene sets ($N = 323$). Multiple testing correction was carried out with **(a)** the function `p.adjust` from the `stats` package setting the argument `method="BH"`, and **(b)** with the respective built-in FDR correction of GSEA and SAFE.

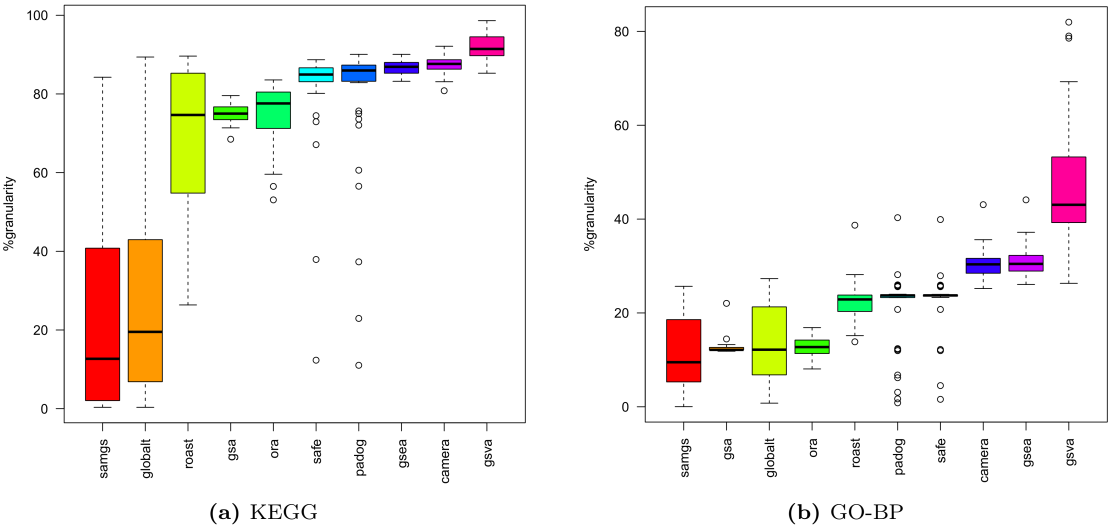

(a) KEGG **(b)** GO-BP

Figure S10: Ranking granularity. Percentage of gene sets with unique p-value returned by SBEA methods when applied to the 42 datasets of the microarray benchmark set and using **(a)** KEGG ($N = 292$), and **(b)** GO-BP ($N = 4128$) gene sets, respectively.

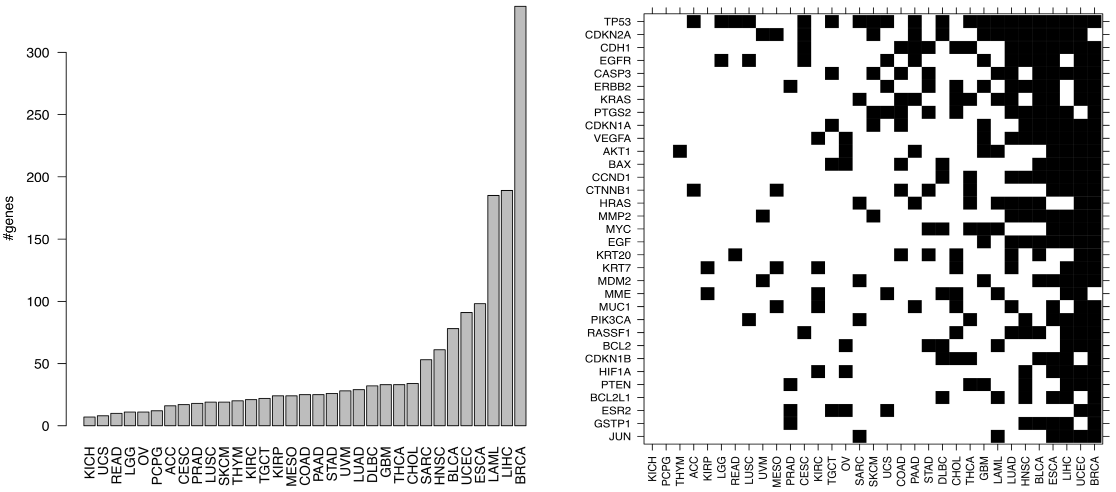

(a) Number of genes **(b)** Most frequent genes

Figure S11: MalaCards disease genes.

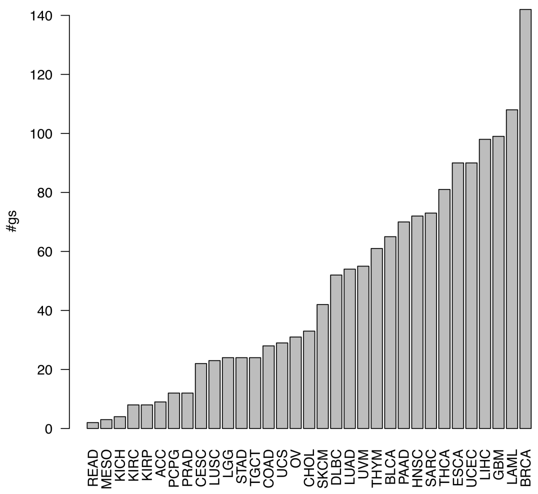

(a) KEGG: number of gene sets

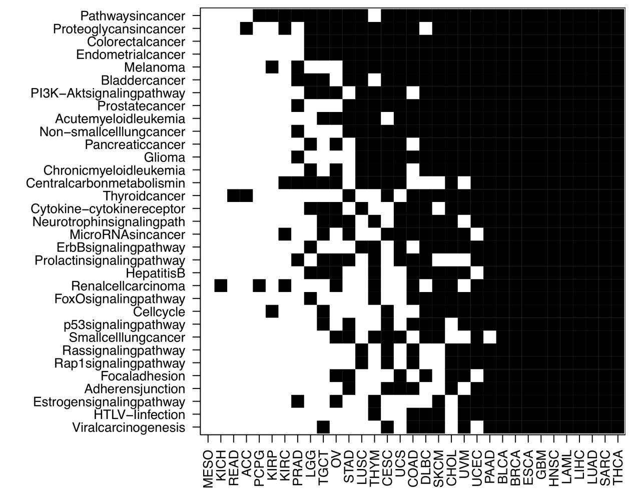

(b) KEGG: most frequent gene sets

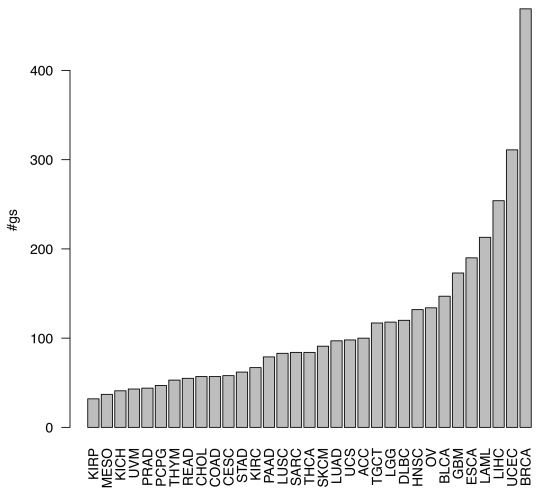

(c) GO-BP: number of gene sets

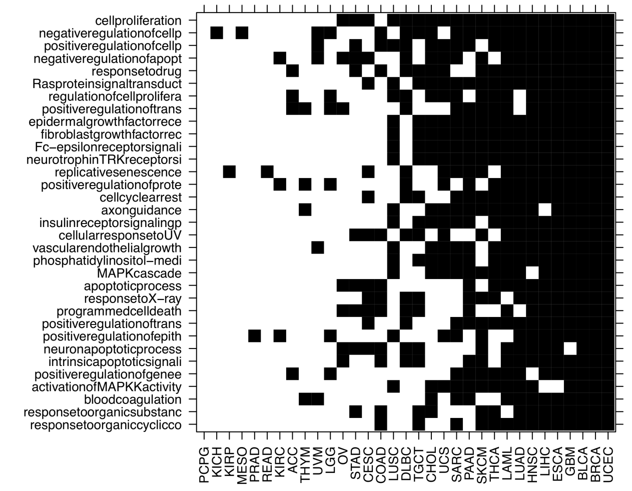

(d) GO-BP: most frequent gene sets

Figure S12: MalaCards disease gene sets.

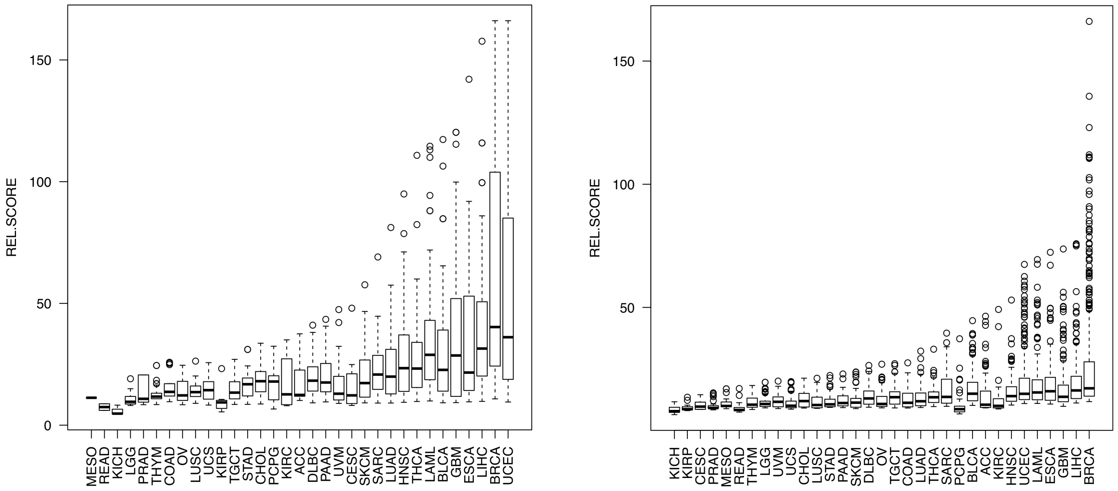

(a) KEGG

(b) GO-BP

Figure S13: MalaCards relevance score range.

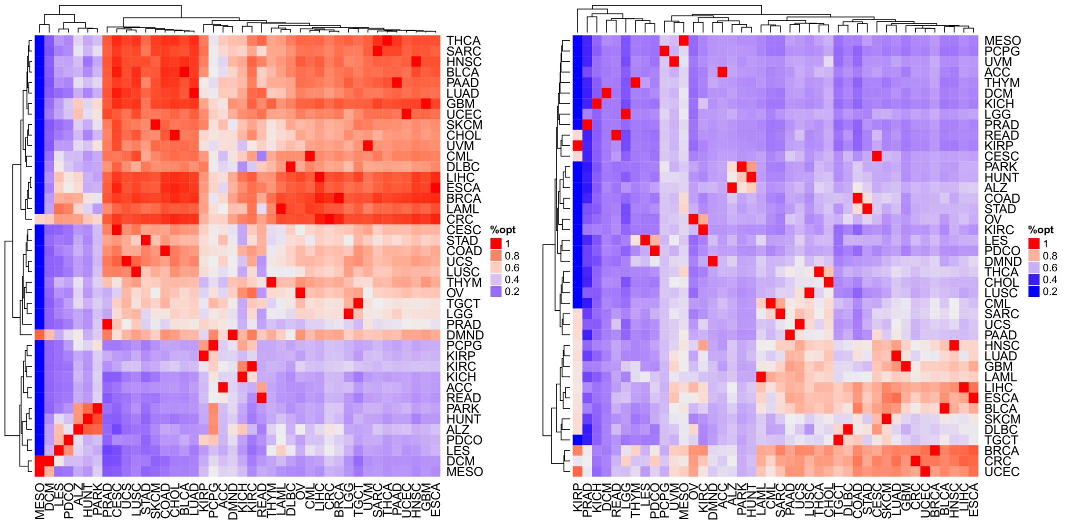

(a) KEGG

(b) GO-BP

Figure S14: Similarity of MalaCards relevance rankings. The heatmap shows the percentage of the optimal phenotype relevance score on a color scale. The optimal relevance score corresponds to the case that two relevance rankings are identical. The heatmap in **(a)** for KEGG shows high similarity between the relevance rankings for cancer types (large red cluster in the upper right), neurodegenerative diseases (ALZ, PARK, HUNT), and previously linked autoimmune / chronic inflammatory lung diseases (LES / PDCO, [38]). These similarities are also apparent to a lesser extent for GO-BP relevance rankings in **(b)**, demonstrating higher similarity of relevance rankings within disease classes than between disease classes.

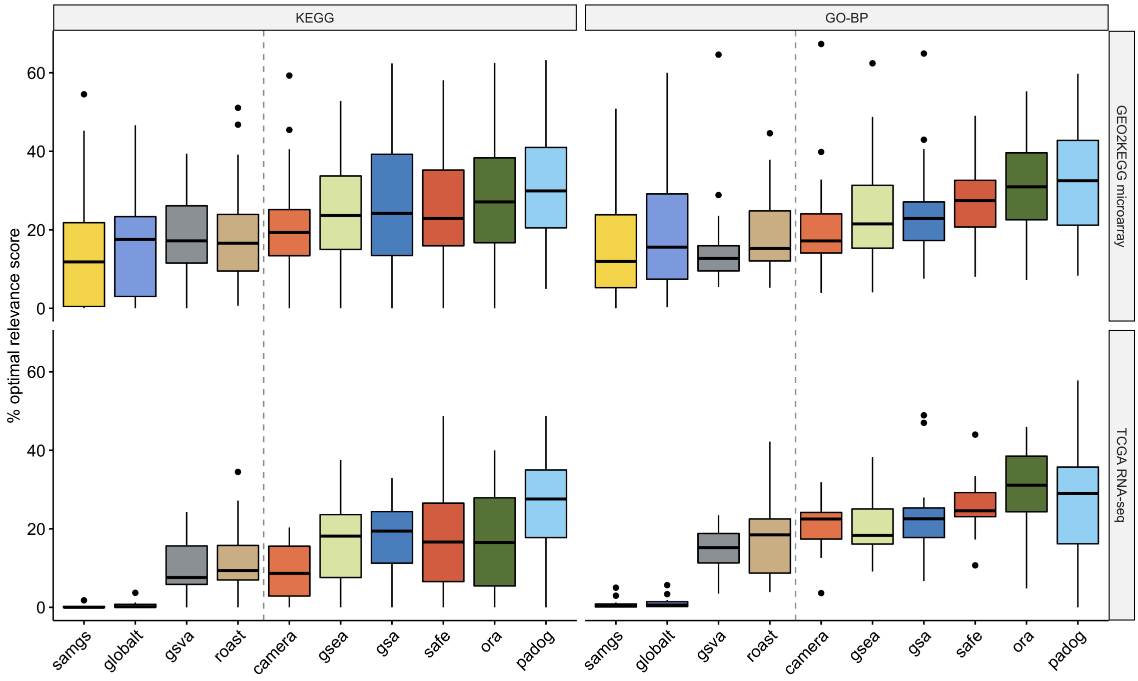

Figure S15: Phenotype relevance for the top 20% of each EA ranking. Percentage of the optimal phenotype relevance score (y-axis) when applying methods to the GEO2KEGG microarray compendium (top, 42 datasets) and the TCGA RNA-seq compendium (bottom, 15 datasets). Gene sets were defined according to KEGG (left, 323 gene sets) and GO-BP (right, 4,631 gene sets). The grey dashed line divides methods based on the type of null hypothesis tested. Computation of the phenotype relevance score is outlined in Figure 1 of the main manuscript and detailed in Methods, Section *Phenotype relevance*.

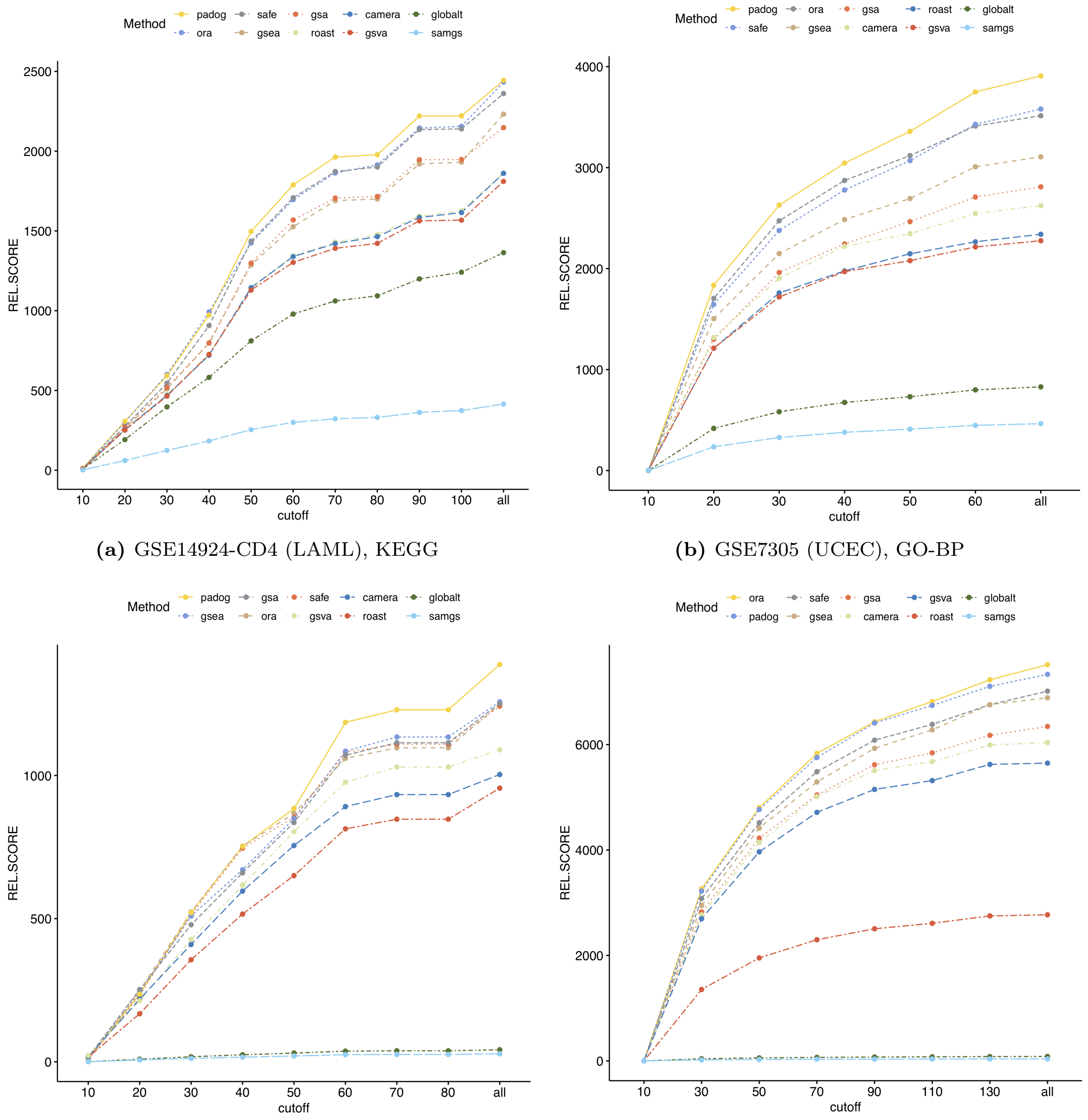

(a) GSE14924-CD4 (LAML), KEGG

(b) GSE7305 (UCEC), GO-BP

(c) THCA (THCA), KEGG

(d) BRCA (BRCA), GO-BP

Figure S16: Accumulation of relevance score at different thresholds. Shown is the phenotype relevance score $X_{m(d)}$ (y-axis) for individual datasets at varying thresholds of the MalaCards relevance score (x-axis).

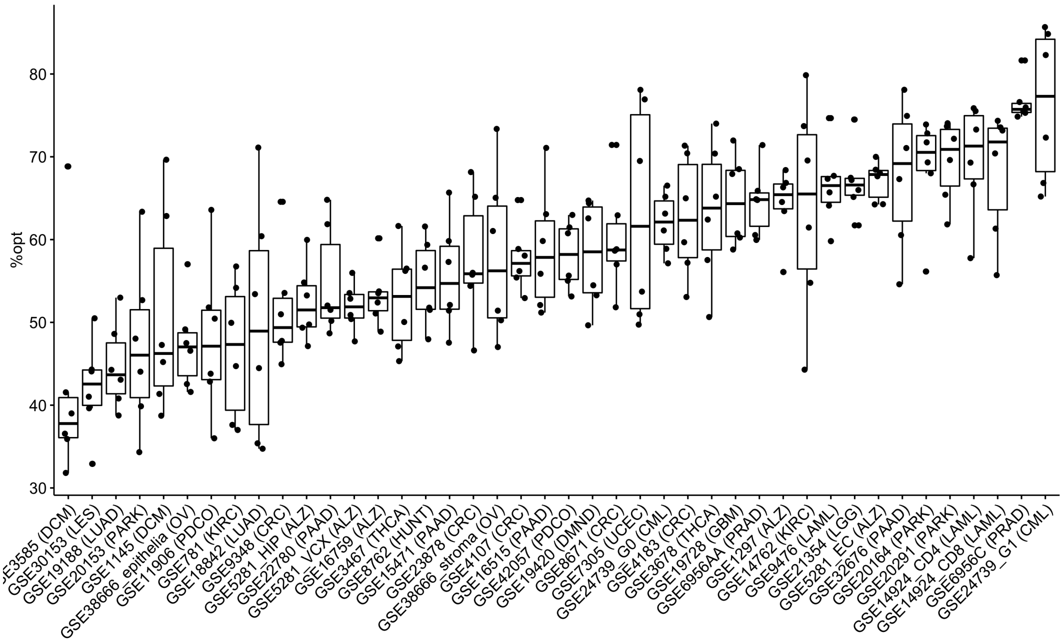

(a) KEGG

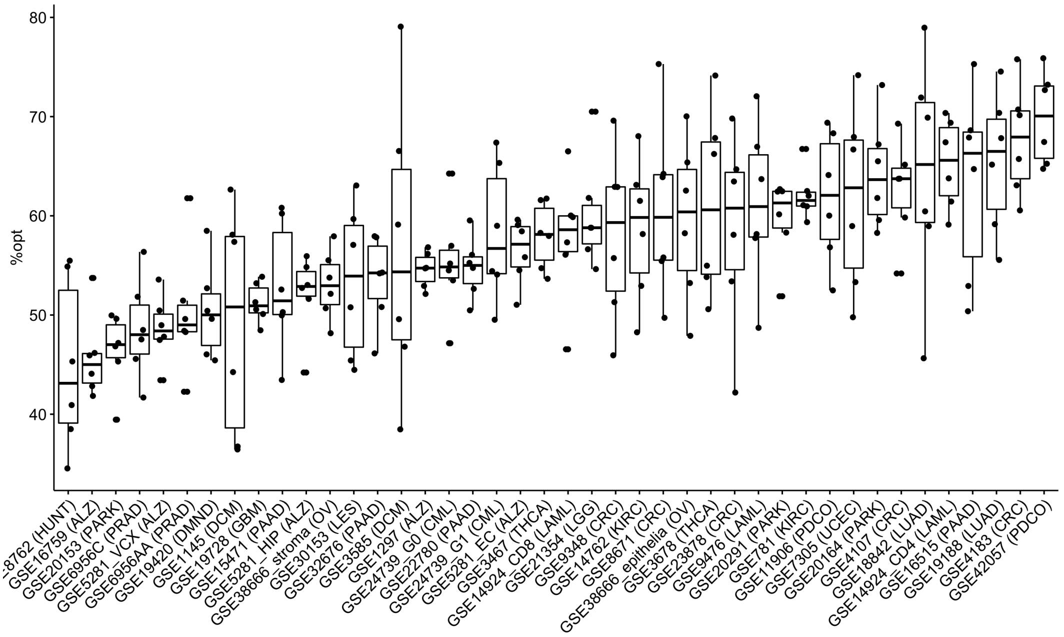

(b) GO-BP

Figure S17: Relevance score distribution per dataset (GEO2KEGG). Shown is the percentage of the optimal phenotype relevance score (y-axis) obtained for the 6 competitive methods (PADOG, ORA, SAFE, GSEA, GSA, CAMERA) for each dataset of the GEO2KEGG microarray compendium (x-axis) for **(a)** KEGG pathways and **(b)** GO-BP terms.

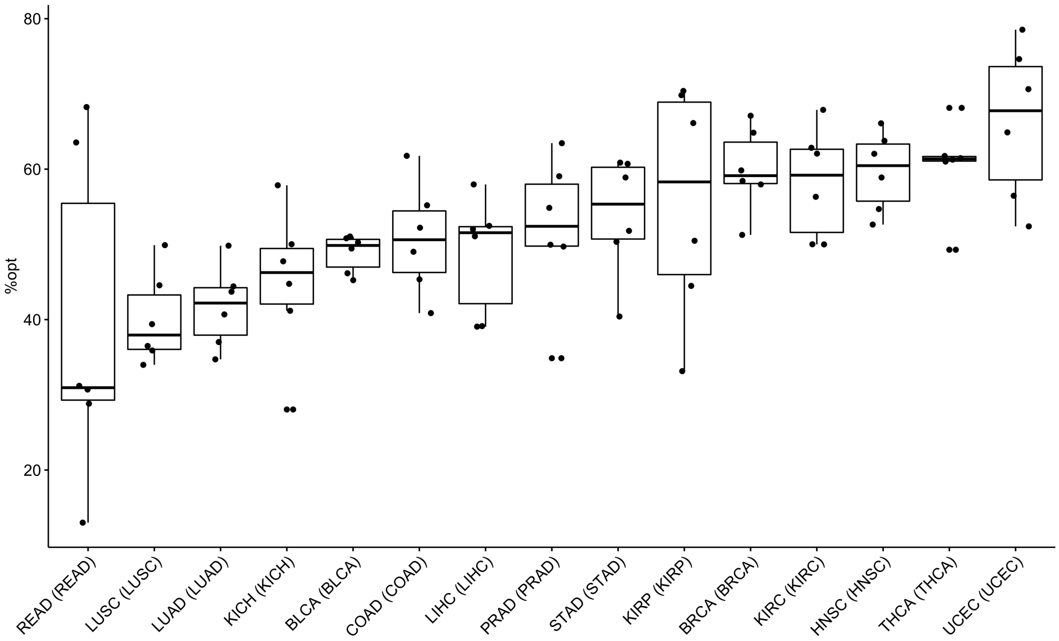

(a) KEGG

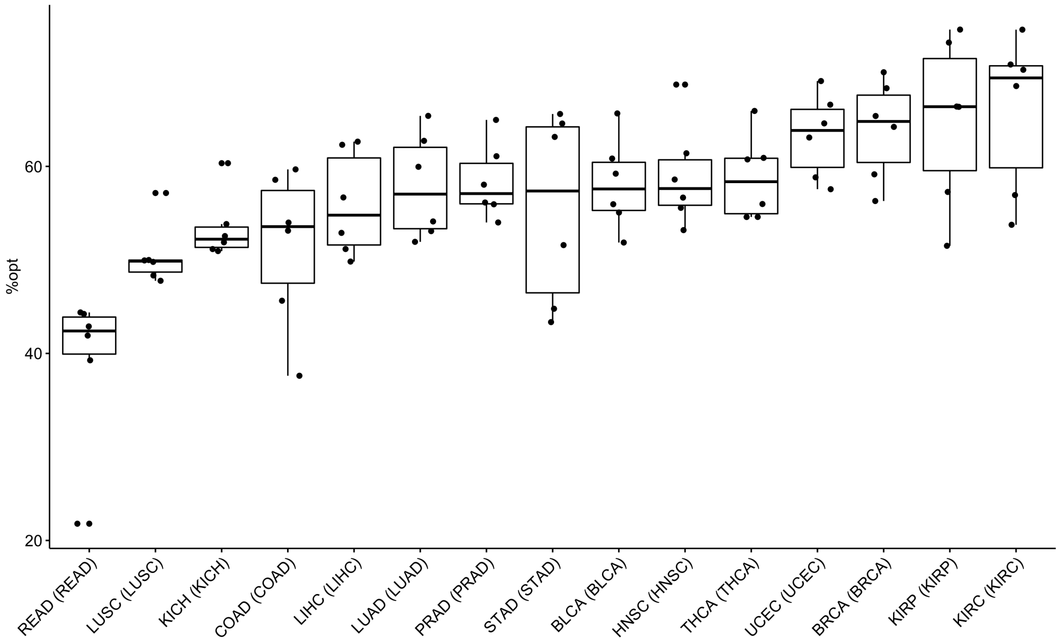

(b) GO-BP

Figure S18: Relevance score distribution per dataset (TCGA). Shown is the percentage of the optimal phenotype relevance score (y-axis) obtained for the 6 competitive methods (PADOG, ORA, SAFE, GSEA, GSA, CAMERA) for each dataset of the TCGA RNA-seq compendium (x-axis) for **(a)** KEGG pathways and **(b)** GO-BP terms.
